# Supplementary material for: Genetic diversity fuels gene discovery for tobacco and alcohol use
Source: Nature. 2022 Dec 7;612(7941):720–4. doi: 10.1038/s41586-022-05477-4 (PMC9771818; doi:10.1038/s41586-022-05477-4)
Supplement: Supplementary file 1 — This file contains Supplementary Notes, Supplementary Figs. 1–5, Supplementary References, and Acknowledgements – see Contents page for details. [file 41586_2022_5477_MOESM1_ESM.pdf]

nature portfolio

https://doi.org/10.1038/s41586-022-05477-4

# Supplementary information

# Genetic diversity fuels gene discovery for tobacco and alcohol use

In the format provided by the authors and unedited

**Supplementary Information to accompany: "Genetic diversity fuels gene discovery for tobacco and alcohol use"**

Saunders *et al.* for the GWAS & Sequencing Consortium of Alcohol and Nicotine use

*Introduction* 3
*Phenotypes* 3
*Study inclusion and generation of summary statistics* 4
Generation of individual summary statistics and ancestry considerations 4
Per-study quality control 6
*Creation of ancestry-specific reference panels from TOPMed* 6
Ancestry specific TOPMed reference panels 7
*Genome-wide association meta-analysis methods* 9
Fixed-effects meta-analysis methods 9
Multi-ancestry meta-analysis methods 9
Region definition and conditional analysis methods 10
Allelic effect size moderation methods 10
Locus definition and fine mapping methods 11
*Genome-wide association meta-analysis results* 12
Post-meta-analysis filters and quality control 12
Ancestry specific meta-analysis and conditional analysis results 12
Multi-ancestry meta-analysis and conditional analysis results 12
Quality control and manual review of all significant loci 13
Replicability of sentinel variants 13
Allelic effect size moderation results 15
Fine-mapping results 16
Functional enrichment 17
*Multi-ancestry TWAS* 17
*Heritability and genetic correlation* 19
*Polygenic scoring* 21
Score construction methods 21
Score prediction accuracy 21
Bias induced by discovery sample and target sample ancestry mismatch 22
*Contributions and acknowledgements* 22

**Detailed author contributions** 22
**Cohort-level acknowledgements** 23
**Individual acknowledgements** 33
***List of Supplementary Figures*** *34*
Supplementary Figure 1. Diagram of project workflow. 35
Supplementary Figure 2. Multi-ancestry meta-analysis QQ plots. 36
Supplementary Figure 3. TOPMed reference panel comparisons. 37
Supplementary Figure 4. Replicability Assessment in Trans-Ethnic Studies (RATES) results for 17 independent variants with low posterior probabilities. 38
Supplementary Figure 5. Affinity propagation clustering of correlations between EUR-stratified GWAS meta-analysis results and 1,141 UK Biobank phenotypes. 42
***References*** *43*

# Introduction

This supplementary text expands on the main text by providing additional explanation, technical details, and fine-grained results. The overall workflow of this study, including its application of standard and novel analytical methods, are displayed in **Supplementary Figure 1**.

# Phenotypes

Phenotypes were selected to represent different stages of substance use across two commonly used licit substances: tobacco and alcohol. Five such phenotypes were widely available across participating studies: four measures of smoking including whether and when an individual begins smoking (smoking initiation and age of initiation of smoking), usual amount smoked among smokers (cigarettes per day), and whether an individual is a current or former smoker (smoking cessation); and one measure of alcohol use (drinks per week). Whenever possible, measures of smoking include cigarette smoking only, as use of other nicotine delivery types were rarely reported across studies. The one exception was for the Amish cohort in the Trans-Omics for Precision Medicine (TOPMed) project[1], as nearly all individuals in that study smoked small cigars. Phenotypes were defined as reported in our previous work[2].

### Smoking Initiation (SmkInit)

1. Binary phenotype with any participant reporting ever being a regular smoker in their life (current or former) coded “2”, while any participant who reported never being a regular smoker in their life coded “1”.
2. Does not include information about pipes/cigar/chew, or other non-cigarette forms of tobacco use.
3. This phenotype was measured in a variety of ways.
    a. *Have you smoked over 100 cigarettes over the course of your life?*
    b. *Have you ever smoked every day for at least a month?*
    c. *Have you ever smoked regularly?*

### Age of Initiation of Regular Smoking (AgeSmk)

1. Age (in years) at which an individual started smoking cigarettes regularly
2. Does not include information about pipes/cigars/chew, or other non-cigarette forms of tobacco use.
3. Measured in a variety of ways:
    a. *At what age did you begin smoking regularly?*
    b. *How long have you smoked?* Combined with *What is your current age?*

### Cigarettes per Day (CigDay)

1. Defined as the average number of cigarettes smoked per day, either as a current smoker or former smoker. Individuals who either never smoked, or for whom there is no available data (e.g., someone was a former smoker, but for whom former smoking was never assessed) were set to missing.
2. For studies that collected a quantitative measure of cigarettes per day, where the respondent is free to provide any integer (e.g., 13 cigarettes per day) responses were binned as follows.
    a. 1 = 1–5
    b. 2 = 6–15
    c. 3 = 16–25
    d. 4 = 26–35
    e. 5 = 36+
3. For studies with pre-defined bins, the pre-defined bins were used.
4. Does not include information about pipes/cigars/chew, or other non-cigarette forms of tobacco use.
5. Cigarettes per day was measured with a single question for most contributing studies using, for example:
    a. *How many cigarettes do you smoke per day?*
    b. *How many cigarettes did you smoke per day?*

### Smoking Cessation (SmkCes)

1. Binary phenotype with current smokers coded as “2”, former smokers coded as “1”, and never smokers coded as missing.
2. Does not include information about pipes/cigars/chew, or other non-cigarette forms of tobacco use.
3. Usually measured through a combination of questions, including:
    a. *Do you currently smoke?* and *Have you ever smoked regularly?*
    b. *Do you smoke?* and *Have you smoked over 100 cigarettes in your entire life?*

**Drinks per Week (DrnkWk)**

1. Defined as the average number of drinks a participant reported drinking each week, aggregated across all types of alcohol. If a study recorded binned response ranges (e.g., 1–4 drinks per week, 5–10 drinks per week) we used the midpoint of the range. For example, if an individual reported 1–5 drinks per week, we assume they drank 2.5 drinks per week on average.
2. This was measured in a variety of ways.
    a. *In the past week, how many alcoholic beverages did you have?*
    b. *Thinking about the past year, on the average how many drinks did you have each week?*
3. This phenotype was left-anchored at 1 and log-transformed prior to analysis, in order to prevent outliers from having undue leverage on analyses.

# Study inclusion and generation of summary statistics

The present project involved a major expansion of a previous effort[2] including new sets of summary statistics from studies that had not previously participated. Summary statistics from studies that had previously participated were either directly re-used, or they were updated (e.g., larger N, improved imputation) and re-shared for meta-analysis. The full list of studies is provided in **Supplementary Table 1**.

Data from 28 studies were obtained through the Trans-Omics for Precision Medicine (TOPMed) program[1], a consortium of studies with deep whole genome sequencing, using genotype calls from freeze 8. To ease computational burden for single-variant tests, we generated marginal summary statistics from each TOPMed study individually, which were then included in the meta-analysis. Relevant phenotypic data was either acquired directly from a dbGaP accession or from the study investigators directly and analyzed centrally. In TOPMed, only smoking phenotypes were available for the current analysis.

## Generation of individual summary statistics and ancestry considerations

Documents outlining the phenotype definitions and analysis plan were shared with participating investigators. Local study personnel conducted GWAS according to this plan and shared summary statistics, imputation quality, and other relevant information such as study design or ascertainment, for meta-analysis. Study personnel were asked to impute their genotypes to the Haplotype Reference Consortium[3] (for European ancestries) or 1000 Genomes[4] phase 3 (for non-European ancestries) using an imputation server such as the Michigan Imputation Server[5] (https://imputationserver.sph.umich.edu; the TOPMed reference panel and imputation server were not yet available at the time requests to participating studies were made). A few studies, including 23andMe and UK Biobank, were imputed using local reference panel resources, which were combinations of 1000 Genomes, Haplotype Reference Consortium, and additional markers from the UK10K[6,7]. All studies were imputed using either Minimac or IMPUTE[8].

The analytical plan requested that study analysts use RVTESTS[9], BOLT-LMM[10], or SAIGE[11] to conduct the GWAS. Some groups had in place an existing robust analytical pipeline, which was also acceptable. Standard covariates included sex, age, age squared, and genetic principal components. Contributing study investigators were instructed to determine the number of genetic principal components and include any additional study-specific covariates associated with their particular study (e.g., genotyping batch, site). Studies composed primarily of closely related individuals (for example, family studies) first regressed out covariates, inverse-normalized the residuals as necessary, and then tested additive variant effects under a linear mixed model with a genetic kinship matrix for all phenotypes. Some studies of unrelated individuals followed the same analysis for quasi-continuous phenotypes (AgeSmk, CigDay, DrnkWk), but estimated additive genetic effects under a logistic

model for binary phenotypes (SmkInit and SmkCes). Differences in the analytical choices between samples of related or unrelated individuals, and the use of linear or logistic models, were based on an attempt to strike a balance between methodological and practical concerns. Many participating studies were large, necessitating the use of computationally efficient approaches, like mixed-effects models, that can account for complex family structures through use of kinship matrices as a random effect. This approach has been shown to work well for genetic association studies of binary phenotypes with high sample prevalence[12,13], has been used successfully previously[14,15], and allows ready inclusion of all individuals within a family sample. The per-study statistics were further normalized prior to running the full meta-analysis to help ensure comparability of effect across studies.

For studies composed primarily of a single major genetic ancestral group, the GWAS analysis plan was applied only to that group. For example, studies were often composed primarily of individuals of European ancestries, with a small number (e.g., < 100) individuals of a different major ancestral group. In those cases, summary statistics were provided only for individuals of European ancestries. If a given study was composed of multiple large ancestry groups, two types of summary statistics were shared in some cases: 1) results based on all individuals in the sample regardless of ancestry, resulting in up to five sets of summary statistics, one for each available phenotype; and 2) results stratified by ancestry, resulting in up to 4×5=20 sets of summary statistics, one for each available phenotype/ancestry combination. Throughout this article, we refer to ancestral groups using terminology from the 1000 Genomes Project[4] as follows:

1. “African” (AFR), composed primarily of individuals with admixed African and European ancestries, primarily from the United States and the United Kingdom, and who may variously self-identify as Black, African American, etc.;

2. “American” (AMR), composed of individuals with admixed American, European and African ancestries, primarily from the United States, and who may variously self-identify as Hispanic, Latino/Latina, etc.;

3. “East Asian” (EAS), composed of individuals with East or Northeast Asian ancestries, primarily including individuals from the People’s Republic of China, Japan, and the United States;

4. “European” (EUR) composed of individuals of European ancestries from the United States, Western Europe, and Australia, and who may self-identify as White, European American, etc.

**Supplementary Table 1** provides study names, sample sizes, and genomic controls for each meta-analysis (multi-ancestry as well as stratified AFR, AMR, EAS, and EUR) and phenotype. Additionally, **Extended Data Figure 1a** shows a scatterplot of all studies, along with 1000 Genomes[4], in the allele frequency-based MDS space of components 1–4.

In general, ancestry assignment was conducted by contributing studies themselves, often based on principal components analysis (PCA) projection onto 1000 Genomes. Ancestry stratification in 23andMe was done using a method developed by the company, which is slightly different from other cohorts. Briefly, 23andMe split genomic data into short windows of approximately 300 SNPs. Haplotypes within each window were classified as one of multiple reference populations (reference populations were derived from the Human Genome Diversity Project, HapMap, 1000 Genomes, and 23andMe customers who have reported having four grandparents from the same country). A hidden Markov model was then used to assign probabilities for each reference population. Final ancestry assignment is based on classification thresholds defined by 23andMe.

TOPMed is composed of individual studies which all were sequenced jointly[1], and we used all available genotypes to identify major ancestries within TOPMed studies. To do this, we grouped individuals in freeze 8 of TOPMed (N = 106,612) using 1000 Genomes phase 3 as a reference for the four major continental ancestry groups, or recent admixtures thereof. Both datasets were subsetted to shared common variants (MAF > 1%, variant missingness < 1%) and LD-pruned using PLINK[16] such that, within 100kb windows, variants with a pairwise $r^2 < 0.01$ are retained before shifting 5 variants and repeating the stepwise procedure. LD pruning was conducted on all variants, ancestry notwithstanding, as prior work has indicated that pruning has minimal effect on the LD within 1000 Genomes[17]. Five ancestry groups were created from the 1000 Genomes reference populations: African (ESN, GWD, LWK, MSL, YRI, ACB, ASW), East Asian (CDX, CHB, CHD, CHS, KHV, JPT),

European (CEU, FIN, GBR, IBS, TSI), South Asian (BEB, GIH, ITU, PJL, STU), and American (CLM, MXL, PEL, PUR). Acronyms are defined as by the 1000 Genomes project[4]. We then applied multinomial logistic regression within 1000 Genomes using the first 15 genetic principal components as predictors, and ancestry groups as the predictand. This fitted model was then applied to TOPMed to make ancestry group assignments. Individuals were classified into an ancestry group if they had a probability > 0.80 of assignment to that group. These groups were then used to generate ancestry-stratified summary statistics for meta-analysis. We note that the ancestry assigned using this method does not necessarily match the ancestry, ethnicity, or culture to which an individual self-identifies.

## Per-study quality control

For each contributing study, we inspected the reported phenotypic distributions for outliers or other unusual distributional characteristics. All summary statistics were oriented to human genome build GRCh38. Results on a different build were lifted over using LiftOver (https://genome.ucsc.edu/cgi-bin/hgLiftOver). For each variant, effect direction, reference/alternate allele, and corresponding allele frequency were modified as necessary such that the reference allele matches the reference allele in human reference genome hg38. Minor allele frequency was used to ensure ambiguous alleles were properly oriented to the reference genome within ancestries. Due to the complexity of calling and coding for insertions and deletions, they were not aligned to any reference dataset. We generated and inspected summary information including allele-frequency-stratified QQ plots, Manhattan plots, genomic controls, and positive control loci (e.g., *CHRNA5* and *CYP2A6* for CigDay; *ADH1B* and *ALDH2* for DrnkWk). For smaller to medium (N < ~10,000) sets of results, we removed studies where the genomic control value based on common variants MAF>.01 was greater than 1.1 or less than .9. For larger studies, we did not apply a max threshold, as true polygenic signal may result in larger genomic controls[18]. No larger study had a genomic control less than .9.

Variants with MAF <= 0.1% were removed from all sets of summary statistics due to low expected imputation accuracy. Imputation for individuals with non-European ancestry performs less well because 1000 Genomes phase 3[4] is the largest reference panel available at the time this work was completed, and so a stricter MAF filter of 1% was applied to studies of primarily non-European ancestry. In all sets of summary statistics, we excluded variants with imputation quality less than 0.3.

# Creation of ancestry-specific reference panels from TOPMed

GWAS analyses were based on summary statistics from participating studies. Many of these analyses benefited from the use of a reference panel with individual-level genotypes to approximate linkage disequilibrium among neighboring genetic variants. For studies of EAS and EUR ancestries, this is relatively simple using either HapMap[19] or 1000 Genomes[4], which have large samples of individuals matched closely to these ancestries. For individuals with more recent ancestry admixture, such as individuals who frequently self-identify as African-American or Latino/a, large sequenced reference panels have not been readily available. To create ancestry-specific TOPMed reference panels that match as closely as possible the ancestry of non-TOPMed studies we considered two options: 1) using all available TOPMed individuals of the same continental ancestry; and 2) selecting a reference sample of TOPMed individuals, regardless of classified continental ancestry, such that the selected centroid of this sample in MDS space is as close as possible to each target non-TOPMed study's MDS centroid. We compared the results of both approaches in TOPMed to each other, and to the use of 1000 Genomes reference panels.

Following the same procedure for generation of TOPMed summary statistics described above, we grouped individuals available from freeze 8 of TOPMed using 1000 Genomes phase 3[4] as a reference for the four major continental ancestry groups, or admixtures thereof. To further improve ancestry group assignment in TOPMed for creation of linkage disequilibrium (LD) reference panels, we used the Online Augmentation Decomposition Procrustes transformation (OADP), wherein the PCs of each individual within TOPMed were mapped to the 1000 Genomes reference PC space using a Procrustes transformation, as implemented in the Fast and Robust Ancestry Prediction by using Online singular value decomposition and Shrinkage Adjustment (FRAPOSA)

software[20]. Visual inspection of the 1000 Genomes PC space identified five extreme outliers from the AFR group, who were removed prior to further analysis (individual IDs HG01880, NA19625, NA20274, NA20299, NA20314). **Extended Data Figure 1b** shows the projection of OADP transformed PCs of 1000 Genomes individuals onto TOPMed individuals. We then evaluated and compared two methods of ancestry prediction based on 1000 Genomes: multinomial logistic regression and *k*-nearest neighbor clustering, both applied to the first 15 OADP PCs from 1000 Genomes with continental ancestry group as outcome. First, the fitted multinomial model was used to predict the ancestry groups of the TOPMed participants. Individuals were classified into an ancestry group if they had a probability > 0.99 of assignment to that group, otherwise, they were set to missing; 1,785 individuals (~1%) were not assigned for this reason. For comparison, a *k*-nearest neighbor algorithm was used as implemented in FRAPOSA with *k* = 20 and weights set to uniform. Individuals were classified into an ancestry group if they had a probability > 0.875 of assignment to that group, otherwise, they are set to missing; 9,034 individuals were not assigned for this reason.

There was substantial agreement in ancestry classification between the multinomial regression approach and the *k*-nearest neighbor algorithm. That is, for TOPMed individuals with a prediction probability above 0.99 for the multinomial regression model and above 0.875 for the *k*-nearest neighbor algorithm, meaning they were classified using both methods, the two classification methods disagreed less than 0.5% of the time (N = 522 individuals were assigned different ancestries across methods). The major difference between the multinomial regression classification and the *k*-nearest neighbor approach is in assignment of individuals with relatively recently admixed ancestry. Despite a much more liberal decision threshold (probability > 0.875 for *k*-nearest neighbor versus probability > 0.99 for multinomial regression) the *k*-nearest neighbor algorithm still resulted in far more unassigned individuals (N = 9,034), as compared to multinomial regression (N = 1,785 individuals unassigned). Because ancestry is continuous, and because a major goal of the reference panel creation is in matching, to the extent possible, individuals of AFR or AMR ancestries, leaving such a large group of individuals unassigned would result in more poorly matched reference panels, particularly for studies with large numbers of individuals of AFR and AMR ancestries. Given these reasons, and to be consistent with the generation of summary statistics in TOPMed, we moved forward with the multinomial logistic regression method, and assigned TOPMed individuals to one of the major continental ancestry groups.

Next, we evaluated the extent to which the TOPMed ancestry groups were comparable with the ancestries represented in all other studies that contributed summary statistics. To make these two data sources commensurate, we projected the TOPMed principal components (based on individual-level genotypes) onto the ancestry space defined by multidimensional scaling of allele frequencies from all studies that contributed summary statistics to our meta-analytic effort. This process involved several steps. First, we took the first three MDS components from allele frequencies for all TOPMed studies of AFR or AMR ancestry (19 AFR TOPMed studies and 8 AMR TOPMed studies), as well as their PC centroids based on the first three principal components. Second, we used a Procrustes transformation to map the matrix of PCA centroids to the matrix of MDS components, such that the squared distance between the two matrices was minimized. This produced a rotation matrix, translation vector, and scale factor that were then applied to the three principal components in the full TOPMed sample (three components were selected because the fourth and greater components essentially distinguished only one study form the rest). We were then able to make comparisons of all TOPMed individuals and non-TOPMed studies in the same space, computing a distance matrix for all points (**Supplementary Figure 3a**).

## Ancestry specific TOPMed reference panels

Once TOPMed individuals (based on PCA) and per-study study summary statistics (based on MDS) were in the same multidimensional space as described above, we compared two approaches to creating reference panels. In the first approach, for each study, we simply selected all TOPMed individuals from the closest TOPMed ancestry (e.g., for 23andMe AFR we used all individuals in TOPMed classified as AFR from our multinomial regression approach). This approach has the dual benefit of being extremely simple and replicable while maximizing the sample size for each reference panel created. We compared this approach to a more involved one, which identified, for each study, a reference panel tailored as closely as possible to the ancestral characteristics of that study. This is advantageous, for example, for conditional analyses[21] that benefit from a tailored reference panel for each contributing study in the meta-analysis. To create such tailored panels, we

iteratively selected TOPMed individuals from any ancestry group, one at a time, such that the allele-frequency-based MDS centroid of the selected TOPMed individuals was as close as possible to the target study MDS location. More specifically, after the single closest (by Euclidean distance) TOPMed individual to a target study is found, all possible combinations of centroids are computed with the closest individual plus every other individual one at a time. The distance between the target study and all possible combinations of centroids is computed and the second individual is selected such that the distance between the target study and selected centroid (now based on two individuals) is minimized. This iterative procedure is repeated until a set number of individuals, who may be of multiple ancestries, are selected as the TOPMed reference panel for a given target study. To provide an illustration, we present a picture of this process using HCHS_SOL in **Supplementary Figure 3b-f**.

To evaluate the performance of these alternative approaches, we compared the LD structure of each TOPMed study to the 'matched' reference panel created from the iterative selection of 10,000 TOPMed individuals ("10k selected"), all TOPMed individuals of the same continental ancestry ("all ancestry"), and all 1000 Genomes individuals of the same continental ancestry. Because we have individual-level genotype data for each TOPMed individual as well as TOPMed study-level genotype data, a close similarity in LD structure between the original TOPMed study and the potential reference panel provides a way to directly compare the performance of the two reference panel options. To do this we extracted TOPMed genotype data from chromosome 20 (filtered to MAF > 0.05) for all individuals in a target TOPMed study, termed the 'original sample'. After removing individuals from the target study, we did the same for the two TOPMed reference panel options, termed 'all ancestry sample' and '10k selected sample', as well as for 1000 Genomes. For all four sets of genotype files we then calculated LD ($r^2$) using VCFtools with a LD window of 100kb for comparison. **Supplementary Table 3a** includes descriptive statistics (means and standard deviations of $r^2$ values) and similarity measures between each possible reference panel (i.e., 1000 Genomes, TOPMed all ancestry, and 10k selected TOPMed individuals) and the original TOPMed study individuals (the original sample which functions as the gold standard). We found that the mean and standard deviations of the LD $r^2$ values were similar between the original TOPMed studies and all ancestry sample options, though 1000 Genomes tended to produce the most dissimilar distributions compared to the original samples. We also found that all ancestry and 10k selected reference samples have higher LD $r^2$ correlations with the original TOPMed studies compared to 1000 Genomes, suggesting that the LD structure of each original TOPMed study is better approximated using the TOPMed all ancestry or 10k selected reference samples.

A second comparison of the reference sample matches was performed using conditional and joint analysis in GCTA-COJO[22], a widely-used software tool to conduct conditional genetic association analysis, with the idea that the best reference panel is the one in which conditional analysis results are most similar to those produced using the original study to construct the panel (the gold standard). For each target TOPMed study, GWAS summary statistics for CigDay (or SmkInit if CigDay was not available in a given study) were used as summary-level statistics along with the reference sample options defined above. In addition, we also used each TOPMed study's own participants as a reference panel, which is a gold standard against which the other reference panel options could be compared. We subsetted all summary statistics to chromosomes 15 and 19, as these chromosomes harbor variants with large and reliable associations with CigDay (and SmkInit). For each set of TOPMed summary statistics the top variant, as indicated by minimum *P*-value in a given TOPMed cohort, was identified and a +/-500kb region was extracted around this variant. We then ran conditional analysis in two ways: 1) by conditioning on the same single top SNP for each set of TOPMed summary statistics and reference panel options, and 2) using a stepwise model selection procedure ('cojo-slct'), with a MAF threshold of 0.01 and *P*-value threshold of $5 \times 10^{-3}$, to identify independently associated SNPs within the region for each pair of summary statistics and reference sample files. We use a lenient *P*-value threshold to ensure that at least one independent variant would be identified for each set of summary statistics, thus allowing for better comparisons across the reference panel options. The purpose of these comparisons was to determine if using each reference sample would result in a similar number of independent SNPs identified compared to the original sample gold standard, as well as to assess the similarity across reference samples of conditional beta estimates and *P*-values after conditioning on the same SNP. In this comparison, shown in **Supplementary Table 3b**, we observed that using all TOPMed individuals of the same ancestry performs as well as, or better, than the 10k selected TOPMed reference panel, both of which outperformed the use of 1000 Genomes itself as the reference panel. This effect was most pronounced for chromosome 19 comparisons. Both types of TOPMed reference samples (TOPMed

all ancestry and 10k selected TOPMed individuals) identified a larger number of independently associated SNPs than the original study reference sample, with the 10k selected sample being slightly more similar than the TOPMed all ancestry reference sample, but both were more similar than 1000 Genomes. Lastly, while the distributions of conditional beta estimates and *P*-values were relatively similar regardless of the reference sample option, the correlations and differences in these values between each reference sample option and the original TOPMed study sample showed the greatest similarity using the TOPMed all ancestry reference sample. Given that the TOPMed all ancestry reference samples well approximated the LD structure of the original TOPMed studies and showed very high agreement for conditional analysis, we used for simplicity and improved replicability the TOPMed all ancestry reference samples for further downstream analyses.

# Genome-wide association meta-analysis methods

## Fixed-effects meta-analysis methods

We conducted four standard fixed-effects meta-analyses – stratified meta-analyses for each of the four main ancestry groups – for each of our five phenotypes (20 total fixed-effects meta-analyses) using a genome-wide significance threshold of $P < 5 \times 10^{-9}$. Refer to **Supplementary Table 1** for the list of studies included for each phenotype for each meta-analysis. We used the software package rareGWAMA (https://github.com/dajiangliu/rareGWAMA) for all meta-analyses. The method aggregates Z-scores across studies with $Z_{META} = \frac{\sum_k w_k Z_k}{(\sum_k w_k^2)^{1/2}}$, where $Z_k$ is the $Z$-score statistic in study $k$. The weight $w_k$ is defined by $w_k = \sqrt{N_k p_k (1 - p_k) R_k^2}$, where $p_k$ is the variant allele frequency, and $R_k^2$ is the imputation quality in study $k$. Thus, the meta-analysis is aware of sample size, variance of the variant, and imputation quality ("imputation quality" for TOPMed whole genome sequencing studies were set to 1.0 for all variants).

## Multi-ancestry meta-analysis methods

We conducted the multi-ancestry meta-analyses using MEMO (Mixed Effect Meta-Regression for Optimal Trans-ethnic Meta-analysis) implemented in the rareGWAMA package again using a genome-wide significance threshold of $P < 5 \times 10^{-9}$. The full model can be described as follow:

$$b_{jk} = \sum_{l=0}^{L} C_{lk} \gamma_{jl} + e_{jk} + \epsilon_{jk}, \quad (1)$$

where $b_{jk}$ is the genetic effect for the $j$th variant in the $k$th study, $e_{jk} \sim \mathrm{N}(0, \tau^2)$ is the random effect that captures unexplained heterogeneity, and $C_{lk}$ was the $l$th axis of genetic variation (or MDS component) for the $k$th study with $C_{0k} = 1$. Correspondingly, $\gamma_{jl}$ captures the effect of the $l$th axis of genetic variation for the $j$th variant with $\gamma_{j0}$ as an intercept in the model. Finally, $\epsilon_{jk} \sim \mathrm{N}(0, s_{jk}^2)$ is the random error term and $s_{jk}^2$ is the standard error corresponding to $b_{jk}$.

We fitted a series of nested models within the full model described above (equation 1). First, we fitted the model that only contains an intercept. In other words, we excluded all axes of genetic variation as well as the random effect. The hypothesis test was performed to examine if the intercept was significantly different from zero. Next, we included the first $l$ MDS components in the model. For each successive model, we added one more component up to 4 total MDS components. Hypothesis tests were performed for each model to test whether each $\gamma$ was significantly different from zero. Finally, for the full model, we tested whether all four $\gamma$'s, as well as whether the variances of random effects ($\tau^2$) were significantly different from zero. Because all statistical tests were performed on the same data, which implied a correlation between them, statistical significance for our final model was calculated using Gaussian copula approach to synthesize information from all models. In addition to estimation of effect sizes, $b_{jk}$, and their variances, $s_{jk}^2$, we also performed genomic control (GC) based on minor allele frequency for rare variants (MAF < 1%) for each participating study.

Per study ancestry variation, $C_{lk}$, is calculated using multidimensional scaling (MDS) based on allele frequencies. We defined the genetic distance between 2 studies, i.e. study $k$ and $k'$, with $J$ variants, as:

$$d_{kk'} = \sqrt{\Sigma_j \left(f_{jk} - f_{jk'}\right)^2}, \tag{2}$$

where $f_{jk}$ and $f_{jk'}$ are the allele frequency for the $j$th variant for study $k$ and $k'$, respectively. We used the first four axes of genetic variation (i.e., we set $L = 4$ in equation 1 above; see **Extended Data Figure 1a**). In this way, MDS component 1 largely separated EAS from other ancestries, component 2 separated AFR from other ancestries, component 3 separated northern vs. southern EUR ancestries, and component 4 separated AMR ancestries from others.

Methods for genetic studies of admixed populations is an area of research under active development[23–25]. The current multi-ancestry GWAS meta-analysis method, which uses cohort-level information, can be viewed as complementary to other recent approaches for admixed samples using local ancestry. Tractor[23], for example, uses individual-level data in a regression model with local ancestry as a covariate to generate GWAS summary statistics. The use of Tractor, or similar methods, by individual cohorts for inclusion in the overall multi-ancestry meta-analysis may be useful in estimating effect sizes in future work.

## Region definition and conditional analysis methods

In reporting the number of regions identified for all meta-analyses, we defined 1MB regions surrounding every genome-wide significant variant, collapsing any overlapping regions into a single region. We used a stringent genome-wide significance threshold of $P < 5 \times 10^{-9}$ for all meta-analyses because we included variants with MAF down to 0.01 (or 0.001 for EUR-stratified results) and variants that might be exclusive to a single ancestry resulting in a greater number of independent tests.

We performed sequential forward selection to identify independent variants for each region as defined above. Specifically, we initialized the set of independently associated variants (denoted by Φ), starting with the top association signal in the region. For each iteration, conditioning on variants in Φ, we performed conditional association analyses for all remaining variants. If the top association signal after the conditional analysis remained significant, we added the top variant to the set Φ, and then repeated the conditional association analysis. If the top variant after the conditional analysis was no longer significant, we stopped and reported variants in the set Φ as the final set of independent variants for that region. We used the same single variant significance threshold ($P < 5 \times 10^{-9}$) as in the marginal meta-analysis to determine statistical significance with the sequential forward selection results and imposed a limit of 10 independent variants per locus (beyond approximately 10 iterations, the stability of LD approximated from the external reference panel declines significantly). Unlike existing conditional meta-analysis methods that use only final meta-analytic results (e.g., genome-wide complex trait analysis; GCTA-COJO[22]), here we make use of study-level summary statistics. Therefore, our method is better able to estimate correlations between score statistics than existing methods when contributing summary association statistics contain missing values, as described previously[21].

Conditional analysis using summary statistics requires external estimates of non-independence among effects of distinct variants. Non-independence between genetic variants was estimated based on linkage disequilibrium patterns for each contributing study estimated from the TOPMed 'all ancestry' reference samples, as described above. This resulted in reference sample sizes of N = 28,665 AFR, N = 19,737 AMR, N = 4,918 EAS, N = 51,656 EUR. Each set of ancestry-stratified fixed-effects results were matched with their relevant reference panel in the analysis (AFR summary statistics with AFR TOPMed panel, AMR with AMR, etc.). For multi-ancestry conditional analyses, we created TOPMed-based reference samples by combining the per ancestry TOPMed reference panels, defined above, resulting in a diverse ancestry reference panel (N = 104,976) cohorts in which to estimate LD.

## Allelic effect size moderation methods

The multi-ancestry meta-analysis model, including the first four per study MDS components, was used to identify variants that showed moderation of effect sizes across ancestries. **Extended Data Figure 1a** shows MDS plots

based on the allele frequencies of each study cohort across phenotypes. Studies are colored by ancestry if they were largely ancestrally homogeneous (>90% of the sample from a single ancestry group), otherwise, studies that contained individuals of multiple ancestries are shown in grey and labeled as 'other' ancestry. We did not stratify studies by ancestry because the multi-ancestry method, MEMO, effectively accounts for this by incorporating MDS components. The first MDS component separates EAS studies, the second MDS component separates AFR studies, the third MDS component separates Northern and Southern EUR studies, and the fourth component separates AMR studies. We used these MDS components to aid in interpretation of multi-ancestry results of allelic moderation across ancestry.

For each independent variant, we evaluated evidence for allelic effect size moderation. In order to do this, the MEMO model was extended into a mixture model representing variants with homogeneous effects (i.e., models with only an intercept term) and those with possible heterogeneous effects (moderation) on at least one axis of genetic variation. Six sub-models were compared: an intercept only (null) model, as well as models that included 0 to 4 MDS components. A likelihood was derived as,

$$L(y) = \prod_{a} p_a^{NULL}\, p\big(b_j|NULL\big) + p_a^{ALT} \sum_{j \in S_a} \left[ q_{j0}\, p\left(b_j \middle| MR_0(j)\right) + \cdots + q_{j4}\, p\left(b_j \middle| MR_4(j)\right) \right], \tag{3}$$

where $p\big(b_j|NULL\big)$ and $p\big(b_j|MR_l\big)$ are, respectively, the likelihoods of the variant $j$ effect sizes under the null model and the meta-regression models with $l$ axes of genetic variation; $p_a^{NULL}$ and $p_a^{ALT}$ are the probabilities of locus $a$ carrying zero or at least one causal variants, respectively. The term $q_{jl}$ is the probability that the model with $l$ axes of genetic variation best fit the data. The model with the largest posterior probability per variant was selected as the best fitting model to capture any genetic effect heterogeneity. Variants in which the model with 0 MDS components was selected were considered to have homogeneous effects across ancestries. Variants in which the selected models had 1 to 4 MDS components were considered heterogenous along the respective axis of genetic variation (e.g., if the selected model for variant $j$ contained 2 MDS components, variant $j$ was considered heterogeneous across component 2, generally indexing an AFR cline). We then took all heterogeneous variants and evaluated the strength of the evidence for effect size moderation. To aid in interpretation, we preferred heterogeneous variants that were polymorphic in two ancestry-stratified meta-analyses. For example, if the MEMO model including MDS component 4 fit best for variant $j$, that variant existed in 2+ ancestry-stratified meta-analyses, and $\gamma_{j4}$ was significantly different from zero, then we considered variant $j$ to be "strongly" heterogeneous on component 4 (AMR GWAS). The significance threshold of $\gamma_{j4}$ was .05 with a Bonferroni correction for the total number of heterogeneous variants for a given phenotype. While we grouped ancestry into four main categories for analytic purposes, genetic ancestry (as shown in **Extended Data Figure 1a**) was continuous, largely without clear boundaries to definitively group individuals. Therefore, we caution against interpreting results as representing group differences.

## Locus definition and fine mapping methods

Fine-mapping was conducted in both the EUR-stratified and multi-ancestry meta-analytic results. Because the method used for fine-mapping assumes a single causal variant within each locus, we relied on a reduced locus size definition, based on LD approximated from a TOPMed reference panel. Specifically, we subsetted the TOPMed sample such that the ancestry proportions roughly matched the full GSCAN sample (81% EUR, 9% EAS, 7% AMR, and 3% AFR) to create an LD reference panel (N = 23,033). From this LD reference panel we extracted all variants with MAF > 0.001 that were in the significantly associated regions (N = 1,449) defined in 'Region definition and conditional analysis methods' above. Then, for every independent variant, we found all variants in LD with the target variant ($r^2$ > 0.1), taking the minimum and maximum positions to define a locus around the target variant. If an independent variant did not exist in the TOPMed reference panel, we used +/- 250kb around the variant to define the locus around the target variant. As above, we collapsed any overlapping regions into a single locus. This resulted in a greater number of loci that are more likely to satisfy the assumption of containing a single causal variant. This LD-based procedure is how loci were defined throughout this manuscript and supplementary note.

To conduct multi-ancestry fine-mapping, we selected the best fitting MEMO model (described in the section above) to approximate the Bayes factor for variant $j$ in a locus by

$$\Lambda_j = exp\left[\frac{X_j - (T+1)lnK}{2}\right], \quad (4)$$

Where $X_j$ denotes the chi-squared test statistic for variant $j$, $T$ denotes the number of axes of genetic variation included in the best fitting model (i.e., 0 to 4 PCs), and $K$ denotes the number of studies contributing to the GWAS. Using the approximate Bayes factor, we calculated posterior inclusion probabilities (PIP) per variant as

$$\pi_j = \frac{\Lambda_j}{\sum_i \Lambda_i}, \quad (5)$$

We then derived 90% credible intervals by ranking variants within a locus by their single posterior estimate and selecting variants until the cumulative posterior probability reached 0.90.

For EUR-stratified fine-mapping, we approximated per variant Bayes factors as above with $T = 0$. We derived 90% credible intervals by ranking variants within a locus by their Bayes factor and selecting variants until the cumulative posterior probability reaches 0.90. Multi-ancestry and EUR-stratified fine-mapping results, based on identical loci, were compared to better describe the resolution gains attributable to inclusion of diverse genetic ancestries.

# Genome-wide association meta-analysis results

## Post-meta-analysis filters and quality control

To help ensure that results were not driven solely by one or two studies, meta-analytic results were filtered for variants that were polymorphic in at least three studies and had an effective sample size >= 0.01 of the maximum sample size for that analysis. The effective sample size is defined as $N_{eff} = \sum_k N_k r_k^2$, where $N_k$ is the sample size in study $k$ and $r_k^2$ is the imputation quality. We filtered out variants with MAF < 0.001 in the multi-ancestry and EUR stratified meta-analysis; we filtered out variants with MAF < 0.01 for AMR-, AFR-, and EAS-stratified meta-analyses.

## Ancestry specific meta-analysis and conditional analysis results

Ancestry stratified meta-analytic results, including all independent variants, are shown in **Supplementary Table 2**. Collapsing across phenotypes, we identified 1,300 regions (2,562 independent variants) in EUR stratified results, 18 regions (36 independent variants) in EAS stratified results, 3 regions (3 independent variants) in AFR stratified results, and 29 regions (32 independent variants) in AMR stratified results. Using results from the ancestry-stratified conditional analysis and a reduced locus definition (detailed in 'Locus definition and fine-mapping methods' section above), we discovered a total of 1,918 loci in EUR stratified results, 19 in EAS stratified results, 3 in AFR stratified results, and 29 in AMR stratified results.

## Multi-ancestry meta-analysis and conditional analysis results

Multi-ancestry meta-analytic results, including all independent variants, are shown in **Supplementary Table 2**, with Manhattan and QQ plots in **Extended Data Figure 2** and **Supplementary Figure 2**, respectively. In multi-ancestry models, we identified 738 regions (2,486 independent variants) for SmkInit, 33 regions (39 independent variants) for AgeSmk, 138 regions (243 independent variants) for CigDay, 132 regions (206 independent variants) for SmkCes, and 408 regions (849 independent variants) for DrinkWk. Using results from the multi-ancestry conditional analysis and a reduced locus definition (detailed above), we discovered 1,346 for independent loci SmkInit, 33 for AgeSmk, 140 for CigDay, 128 for SmkCes, and 496 for DrnkWk. In total, multi-ancestry results identified 3,823 independent variants, an increase of 1,190 variants (45.2%) over ancestry-stratified conditional analysis results.

To evaluate the increase in power by modeling genetic effect heterogeneity as in our multi-ancestry approach over that of simpler models, we compared the number of loci identified in the full multi-ancestry meta-regression results to those identified using models with identical data but without consideration of ancestry (essentially the fixed-effects MEMO sub-model without including per study MDS components or the random effect term). This

comparison yielded 50 more loci identified in the multi-ancestry results (a 2.4% increase). This illustrates the increased power to identify associated loci in our meta-regression random effects approach.

## Quality control and manual review of all significant loci

We evaluated meta-analytic Cochrane's Q and $I^2$ statistics to evaluate effect heterogeneity across contributing studies for all independent variants within each locus for each set of ancestry stratified and multi-ancestry results. Q was considered significant after application of a Bonferroni correction for all tests (i.e., all independent variants) within a phenotype.

Across phenotypes, 25 variants showed heterogeneity per the Q statistic in the EUR-stratified results, ten variants in EAS-stratified results, and two for AMR-stratified results. No variants showed heterogeneity in AFR-stratified results. These variants are still reported and remained in downstream analyses, but caution is advised in the interpretation of their effect. Heterogeneity statistics are reported for all independent variants in **Supplementary Table 2**.

All multi-ancestry genome-wide significant regions were plotted with LocusZoom, were manually reviewed, and regions with apparently odd association patterns (e.g., no LD support within the locus) evaluated in detail. LocusZoom images were made using the LocusZoom standalone software (https://genome.sph.umich.edu/wiki/LocusZoom_Standalone) using TOPMed ancestry matched reference samples for LD information (the N=104,976 TOPMed sample described in the 'conditional analysis' section above) and the UCSC genome browser for dbSNP and gene positions. We set the most significant variant in the region as the reference variant upon which LD in the window is based. We report heterogeneity statistics in **Supplementary Table 2** for multi-ancestry results, finding 43 variants with significant Q statistics. This is not unexpected given that many study-level summary statistics were stratified by ancestry before being included in the multi-ancestry models, and we are specifically interested in testing whether variant effect sizes differ by ancestry.

## Replicability of sentinel variants

In all meta-analyses, we applied genomic control (GC) correction for low frequency variants (MAF < 1%). GC correction for common variants was not applied because high polygenic-based inflation of test statistics is expected[18], especially with large sample sizes. We surmised that a strict GC control would be overly conservative. To evaluate this decision, we assessed the robustness of our results and the extent to which they are affected by population stratification in three ways.

First, we used a novel statistical method (Replicability Assessment in Trans-Ethnic Studies; RATES) to assess replicability of each multi-ancestry sentinel variant. RATES is a trans-ancestry extension of MAMBA[26] (Meta-Analysis Model-based Assessment for replicability), a method for assessing the posterior probability of replicability of associations without the need for an independent replication sample. RATES leverages the strength and consistency of associations across cohorts, by incorporating study-level summary statistics and per-study allele frequency MDS components. For a given variant, RATES assigns a posterior probability by:

$$PPR_j = \hat{P}\left(R_j = 1 \middle| \boldsymbol{b_j}, \widehat{\boldsymbol{\Psi}}\right) = \frac{P\left(R_j = 1 \middle| \widehat{\boldsymbol{\Psi}}\right) p\left(\boldsymbol{b_j} \middle| R_j = 1, \widehat{\boldsymbol{\Psi}}\right)}{p\left(\boldsymbol{b_j} \middle| \widehat{\boldsymbol{\Psi}}\right)}, \quad (6)$$

where $\boldsymbol{b_j}$ is a vector of effect size estimates across $k$ studies for variant $j$. $R_j$ is an indicator variable denoting a variant with replicable effect, and $\widehat{\boldsymbol{\Psi}}$ is the hyperparameter estimate for the fitted RATES model based on the observed summary statistics.

In the current analysis, we prepared per-study summary statistics (variant effect size estimates and their standard errors) for all sentinel variants as well as for randomly pruned null effect variants (variants that failed to achieve genome-wide significance and were located outside of associated loci), as well as the first four per-study MDS components, as required by RATES. For each chromosome, we included 2000 null variants, and ran the RATES

model by each chromosome for all five traits. The resulting posterior probabilities then served as a metric for assessing replicability of sentinel variants as well as the loci in which they were contained.

In general, the posterior probability of replicability was high, with only 17 variants (of 1,449) falling below a threshold of < .99. We removed these variants and their associated regions from downstream analyses (i.e., conditional analysis, allelic heterogeneity, fine-mapping). Seventeen variants (and loci) were removed for this reason; two for SmkInit (chr3:38609794, chr6:79798179), two for CigDay (chr2:134785856, chr18:45078216), 10 for SmkCes (chr2:106169084, chr11:79227043, chr15:42576159, chr3:173570664, chr15:26719238, chr2:160587711, chr13:100261629, chr16:15127835, chr13:57909623, chr11:11839580), and three for DrnkWk (chr1:102463444, chr18:43149880, chr18:27674354).

Second, we used LD Score Regression (LDSC)[27] to further probe replicability of results and possible influence of population stratification (**Supplementary Table 8**). We inspected the intercept from LDSC for EUR and EAS ancestries and that from covariate-adjusted LDSC[28] (cov-LDSC) for AMR and AFR ancestries. Detailed procedures for LDSC and cov-LDSC are provided in 'Heritability and Genetic Correlation' section below, including the choice of reference panel. Briefly, we used LD scores estimated in a random subsample of TOPMed reference samples of each ancestry (EUR, AFR, AMR, EAS) after excluding related individuals (< 4th degree). Intercepts that depart substantially from 1 have traditionally been interpreted as evidence for population stratification[27,30]. However, in many empirical applications of LDSC, the intercept often rises above one due to large sample sizes under the assumption of high polygenictiy[10], which we expect is true for the current analysis given our sample sizes yielding a high number of GWAS hits. To mitigate the expected bias, we examined the attenuation ratio, (LDSC intercept – 1) / (mean $\chi^2$ – 1), for each meta-analysis result[10].

In **Supplementary Table 8**, LDSC intercepts for most meta-analysis results were close to 1 (range: 0.99-1.036) except for SmkInit in AMR ancestry and most phenotypes in EUR ancestry (range: 1.07-1.44). For those with elevated intercepts, the attenuation ratios were less than 1, consistent with the notion that these large intercepts are biased due to polygenicity, and that our type I errors were ultimately well controlled with respect to population stratification. The AFR ancestry-stratified analyses for AgeSmk and CigDay had the smallest sample sizes of any phenotype-ancestry combination (N=17,518 and N=20,157, respectively, for the LDSC analyses). This resulted in larger standard error estimates for SNP heritabilities, intercepts, and attenuation ratios than other phenotypes. The attenuation ratio is particularly important for genetic association results based on very large samples (e.g., >500K), where bias is induced in the intercept. Due to the relatively small sample sizes for AgeSmk and CigDay in AFR ancestries caution is warranted in interpretation of the attenuation ratio. Estimates of the genomic control factor and intercept may be more relevant. Taken together, the AgeSmk and CigDay LDSC analyses in AFR-stratified samples may be somewhat underpowered.

Third, we evaluated the effects of stratification using within-family association analyses for N = 15,843 pairs of siblings from the UK Biobank. We regressed the residualized phenotype of each individual within a sibling pair on the deviation of that sibling's genotype from the family mean:

$$\hat{y}_{ij} = \hat{\beta}_{BF}\overline{\mathrm{g}}_{\mathrm{jk}} + \hat{\beta}_{WF}\left(g_{ijk} - \overline{\mathrm{g}}_{\mathrm{jk}}\right) + \varepsilon_{ij}, \tag{7}$$

where $\hat{y}_{ij}$ is the residualized phenotypic value of individual $i$ in sibling pair $j$ after partialling out covariates of sex, age, age$^2$, and the first 20 PCs, $\overline{\mathrm{g}}_{\mathrm{jk}}$ is the mean genotype of variant $k$ in sibling pair $j$, $g_{ijk}$ is the genotype of the $k$th variant of individual $i$ in sibling pair $j$, and $\varepsilon_{ij}$ is the random error.

Due to our small sibling sample size, we were underpowered to discover or even replicate individual variant associations. Instead, following Okbay et al.[31] and Lee et al.[32], we used a simple test of how often the full EUR stratified GWAS results without the UKB cohort and the within-family estimates had concordant signs. For this analysis, we considered only sentinel variants from the EUR fixed-effects meta-analyses that were polymorphic with MAF > 1% in the UK Biobank sibling data (N = 1,278 variants).

For the sign test, the null hypothesis was that all GWAS results are driven entirely by population stratification, cryptic relatedness, or other confounding factors. In this case, the sign of the within-family estimates would be completely independent of the sign of the GWAS estimates, resulting in an expected sign concordance of 50%. To formally test this, we compared the observed sign concordance against the expected sign concordance under

the null hypothesis, which follows a binomial distribution with *n* equal to the number of sentinel variants and *p*, the concordance probability, equal to 0.5. Given that we expect at least some of the GWAS signal to be a true genetic effect, we used a one-sided alternative hypothesis of sign concordance > 50%.

For every phenotype, the observed sign concordance was significantly greater than what we would expect under the null hypothesis that our GWAS results were driven by population stratification. For SmkInit we observed sign concordance of 68.1% (514/755 variants have concordant signs across within-family estimates and GWAS, $P < 2.2 \times 10^{-16}$, 95% CI [.65, 1]); AgeSmk 80% (16/20 variants, $P = 0.006$, 95% CI [.59, 1]); CigDay 63.5% (66/104 variants, $P = 0.004$, 95% CI [.55, 1]); SmkCes 68.3% (56/82 variants, $P = 0.0006$, 95% CI [.59, 1]); and DrnkWk 63.4% (201/317 variants, $P = 1.04 \times 10^{-6}$, 95% CI [.59, 1]). These results are consistent in magnitude with other large-scale association studies[32], suggesting that population stratification is controlled to the same extent as other, similar, studies.

Given the small sample size and reduced power in the within-sibling comparison, we also evaluated effect size sign concordance of sentinel variants based on EUR-stratified 23andMe summary statistics in EUR-stratified summary statistics with all cohorts included except 23andMe. Specifically, within 23andMe summary statistics we defined 1MB regions surrounding every genome-wide significant variant ($P < 5 \times 10^{-9}$), collapsing any overlapping regions into a single region. If applicable, we removed the 17 variants listed above with low posterior probabilities. We then extracted the variant from each region with the smallest *P*-value (i.e., the sentinel or lead SNP) from the EUR-stratified 23andMe summary statistics and the EUR-stratified summary statistics of all cohorts except 23andMe, comparing the fraction of variants with concordant directions of effect. For SmkInit we observed sign concordance of 97.4% (592/608 variants have concordant signs); AgeSmk 100% (2/2 variants); CigDay 100% (26/26 variants); SmkCes 94.3% (33/35 variants); and DrnkWk 94.8% (218/230 variants). All associated *P*-values were less than $1 \times 10^{-16}$.

## Allelic effect size moderation results

Power analysis showed that we have 80% power to detect standardized heterogeneity effects as small as $3.94\times10^{-5}$ for MDS component 1 ($\gamma_1$), $5.15\times10^{-5}$ for MDS component 2 ($\gamma_2$), and 0.0002 for both MDS components 3 ($\gamma_3$) and 4 ($\gamma_4$). This suggests that our tests for allelic heterogeneity were, in general, well powered to detect modest effect size moderation by ancestry. Full allelic heterogeneity results for each independent variant are shown in **Supplementary Table 2**, with a summary of findings for each phenotype below.

For SmkInit, we found 524 variants (21.0% of independent variants) with heterogeneous effects identified based on model selection alone. Among these, 74 variants (3.0% of independent variants) showed strong evidence for allelic heterogeneity: 50 variants were heterogeneous on component 1, 17 variants were heterogeneous on component 2, 2 variants were heterogeneous on component 3, and 5 variants were heterogeneous on component 3.

For AgeSmk, we found 7 variants (17.9% of independent variants) with heterogeneous effects identified based on model selection alone. Among these, 4 variants (10.3% of independent variants) showed strong evidence for allelic heterogeneity: 3 variants were heterogeneous on component 1, and 1 variant was heterogeneous on components 3.

For CigDay, we found 57 variants (23.4% of independent variants) with heterogeneous effects identified based on model selection alone. Among these, 20 variants (8.2% of independent variants) showed strong evidence for allelic heterogeneity: 16 variants were heterogeneous on component 1, and 4 variants were heterogeneous on component 2.

For SmkCes, we found 31 variants (15.1% of independent variants) with heterogeneous effects identified based on model selection alone. Among these, 7 variants (3.4% of independent variants) showed strong evidence for allelic heterogeneity: 4 variants were heterogeneous on component 1 and 3 variants were heterogeneous on component 2.

Finally, for DrnkWk, we found 183 variants (21.5% of independent variants) with heterogeneous effects identified based on model selection alone. Among these, 31 variants (3.6% of independent variants) showed strong evidence for allelic heterogeneity: 15 variants were heterogeneous on component 1, 5 variants were heterogeneous on component 2, 6 variants were heterogeneous on component 3, and 5 variants were heterogeneous on component 4.

We investigated whether our allelic heterogeneity findings were driven by differential imputation quality across ancestries, LD score differences, or population differentiation (Fst values). For every independent variant we computed per ancestry LD scores using all TOPMed cohorts that contributed to the multi-ancestry meta-analysis, calculated the mean imputation quality scores across all contributing cohorts to the SmkInit GWAS for each ancestry, and again using the TOPMed cohorts, computed Fst values for each pair of ancestry groups (i.e., pairwise EUR-EAS, EUR-AFR, EUR-AMR, AFR-AMR, AFR-EAS, and AMR-EAS). We then compared the distributions of each of these (LD scores, imputation quality, and pairwise Fst) between the 3,032 variants with no evidence of heterogeneity and the 136 variants with strong evidence of heterogeneity. We also compared the distributions between variants that were heterogeneous on each MDS components and those with no evidence of heterogeneity (e.g., comparison between the 88 variants showing allelic heterogeneity across MDS component 1 and those with no evidence of heterogeneity on any MDS component).

In total there were 70 mean comparisons with only three *P*-values below the Bonferroni corrected threshold: (1) significantly lower imputation quality in EAS ancestry for variants heterogeneous on MDS component 1 (M = .95, SD = .09) compared to variants with no evidence of heterogeneity (M = .97, SD = .04), $t(111.6) = -5.11$, $P$ = 1.36e-6; (2) significantly greater pairwise Fst between AFR and EAS ancestries for variants heterogeneous on MDS component 3 (M = .12, SD = .14) compared to variants with no evidence of heterogeneity (M = .02, SD = .02), $t(9.94) = 13.95$, $P$ = 7.49e-8; and (3) significantly greater pairwise Fst between AFR and AMR ancestries for variants heterogeneous on MDS component 4 (M = .07, SD = .09) compared to variants with no evidence of heterogeneity (M = .02, SD = .03), $t(9.75) = 5.71$, $P$ = .0002. The small number of mean differences across ancestries and a lack of clear pattern of results suggests that the identification of heterogeneous variants was not driven to a large extent by differential imputation quality, LD scores, or Fst across ancestries.

## Fine-mapping results

We used fine-mapping with the inclusion of diverse ancestries to improve resolution of loci implicated at genome-wide significance. **Supplementary Table 4** includes multi-ancestry fine-mapping results for loci with less than 5 variants in the 90% credible interval.

Consistent with expectations based on prior work[33,34] comparing multi-ancestry to EUR-stratified fine-mapping results, 90% credible intervals in multi-ancestry contained fewer variants and were smaller in size, on average, than EUR-stratified results (average reduction of 33.3% in the median number of variants and 24.3% in the median width). In multi-ancestry fine-mapping, across phenotypes, we found that an average of 27.9% of credible intervals contained less than five variants, and 9% of credible intervals contained a single variant. We found six loci in which credible intervals from the EUR-stratified results contained more than 975 variants. We re-ran comparisons after removing these loci and found the results to be highly similar and substantive conclusions unchanged.

We evaluated to what extent the increased multi-ancestry fine-mapping resolution, over EUR-stratified results, was due to increased sample sizes or increased genetic diversity. We compared fine-mapping in multi-ancestry results to that of ‘downsampled’ multi-ancestry results in which we removed EUR ancestry cohorts until the total sample size was approximately equal to that of the EUR-stratified analysis for each phenotype. We found that in the 1,330 (62.1%) loci more precisely fine-mapped in the full trans-ancestry analysis, the credible intervals were 45.5% smaller in downsampled multi-ancestry results compared to EUR-stratified fine-mapping, suggesting that nearly half of the observed reduction in credible interval size is attributable to differences in sample size while the remainder is attributable to inclusion of diverse ancestries. These results highlight the importance of both increased sample size and inclusion of diverse ancestries.

## Functional enrichment

We combined findings from the multi-ancestry fine-mapping analyses with functional genomics data to test whether high priority genes were enriched in specific tissues, brain cell types, and gene pathways. We defined high priority genes here as those mapped from variants in fine-mapped credible intervals containing less than five variants based on the hg38 UCSC knownGene annotation database. These genes were compared to 'control' genes identified in the same way, but from variants with PIP $< 0.01$ from the multi-ancestry fine-mapping. For variants that were not in a known gene, we assigned the nearest gene. The purpose of this analysis was to evaluate biological characteristics of the genes implicated in well fine-mapped regions compared to genes from variants with little evidence of causal association.

To maximize power for comparison, we combined the high priority gene lists across all five phenotypes, resulting in 583 high priority genes associated with variants from the loci fine-mapped to less than five variants. In 335 of the 583 genes, fine-mapped variants were located within genes (i.e., not intergenic); the rest were located in intergenic regions (mean distance from the nearest gene of 181kb with a standard deviation of 303kb). We then estimated the relative risk of these 583 genes being in several annotation categories related to tissue expression, brain cell type, and specific gene pathways. The estimated relative risk is the ratio of the proportion of high priority genes that are in the annotation category to the proportion of control genes in the same annotation category. Using brain tissue expression as an example, the relative risk estimate would be the ratio of high priority genes enriched in brain tissue to the total number of high priority genes divided by the ratio of control genes enriched in brain tissue to the total number of control genes.

For tissue expression, following previous work[35], we obtained gene sets for the top 10% of enriched genes in 37 GTEx[36] tissue types and then calculated the relative risk for each of the 37 tissues. For cell type comparisons, we obtained gene sets for the top 10% of enriched genes in 39 brain cell types as described in Bryois et al.[35], calculating the relative risk for each brain cell type. Lastly, for gene pathways, we used the C5 (gene ontology) gene sets and calculated the relative risk for each gene pathway. We removed gene sets with fewer than 10 genes and those that did not exist either in our fine-mapped loci nor loci with PIP < 0.01, resulting in 7,115 gene-sets. For each relative risk estimate we constructed 95% confidence intervals and *P*-values with 1,000 replications each for tissue and cell type comparisons, and 100,000 replications for gene pathway comparisons.

For all phenotypes, high priority genes had significantly greater enrichment in all 13 brain tissues compared to control genes after Bonferroni correction. These genes were strongly associated with telencephalon projecting excitatory and projecting inhibitory neurons within the central nervous system. Lastly, high priority genes were enriched for gene ontology terms related to neurogenesis, synapses, and neuron differentiation. Full results are shown in **Supplementary Table 5** and **Extended Data Figure 3**.

# Multi-ancestry TWAS

Multi-ancestry transcriptome-wide association analyses were performed using a novel method, the "trans-ethnic transcriptome-wide association method" (TESLA). TESLA uses a meta-regression similar to MEMO to model phenotypic effects across different studies in a trans-ethnic meta-analysis, accounting for potential genetic effect heterogeneity across ancestry. TESLA then performs TWAS using improved phenotypic effect estimates in the matched ancestry of the eQTL data, which substantially increases power over other TWAS methods. The power advantage increases with diversity of the GWAS data and with the extent of heterogeneity in the phenotypic effects across ancestries.

Specifically, TESLA begins by fitting a series of models ($M^{[L]}$) based on the first $L$ MDS components. Genetic effect estimates for each $M^{[L]}$model are given as:

$$M^{[L]}: b_{jk} = \sum_{l=1}^{L} C_{lk}\gamma_{jl} + \epsilon_{jk}, \quad (8)$$

where $b_{jk}$ is the genetic effect for the $j$th variant in the $k$th study and $C_{lk}$ is the $l$th axis of genetic variation (or MDS component) for the $k$th study. Correspondingly, $\gamma_{jl}$ captures the effect of the $l$th axis of genetic variation for the $j$th variant. Finally, $\epsilon_{jk} \sim N(0, s_{jk}^2)$ is the random error term and $s_{jk}$ is the standard error corresponding to $b_{jk}$. This is similar to the trans-ancestry MEMO model in equation 1 but without the random effect.

Based on meta-regression coefficients (from equation 8), the phenotypic effects in the eQTL dataset are estimated for a series of models from 1 to $L$ MDS ancestry components. This is given by

$$\hat{h}_j^{[L]} = \tilde{C}^{[L]}\hat{\gamma}_j^{[L]}$$

where $\hat{h}_j^{[L]}$ are the estimated phenotypic effects of variant $j$, $\tilde{C}^{[L]}$ are the first $L$ MDS component values of the eQTL dataset, and $\hat{\gamma}_j^{[L]}$ are the estimated effects of the $L$th MDS component from equation 8, all corresponding to the $M^{[L]}$ meta-regression model. From here, we used the vectors of phenotypic effect estimates ($\hat{h}^{[L]} = \left[\hat{h}_1^{[L]}, \ldots, \hat{h}_J^{[L]}\right]$) to construct a TWAS statistic for each $M^{[L]}$ model:

$$U_{TWAS}^{[L]} = \sum_{j=1}^{J} w_j \hat{h}_j^{[L]} / s_j^{[L]}$$

where the weights, $w_j$, are taken from PrediXcan[37] and were trained based upon 49 tissues from GTEx[36] release version 8.

For the current analysis, four MDS components were included resulting in calculation of four different TWAS statistics. We used a minimal p-value approach to find the overall *P*-value for the statistic. Specifically, we denote the *P*-values for the 4 statistics as $P^{[1]}, \ldots, P^{[4]}$. The minimal *P*-value statistic $P^*$ (i.e., $min\left(P^{[1]}, \ldots, P^{[4]}\right)$) follows:

$$\begin{aligned} Pr(P^* < p^*) &= 1 - Pr(P^* > p^*) \\ &= 1 - Pr\left(\Phi^{-1}(1-p^*) < U_{TWAS}^1 < \Phi^{-1}(p^*), \ldots, \Phi^{-1}(1-p^*) < U_{TWAS}^4 < \Phi^{-1}(p^*)\right) \end{aligned}$$

which can be evaluated using multivariate normal distributions. We calculated a combined cross-tissue *P*-value for each gene using the Cauchy combination[38] method assigning equal weight to each tissue. This method is well-suited for combing *P*-values under a correlational structure and is computationally tractable with large quantities of data as we have here.

Using a *P*-value threshold of $4.61 \times 10^{-8}$ (Bonferroni correction for 22,121 genes in 49 tissues), we found 14,028 gene-tissue associations for SmkInit, 177 associations for AgeSmk, 2,912 associations for CigDay, 2,193 association for SmkCes, and 5,729 associations for DrnkWk (**Supplementary Table 6**). Based on multi-tissue TWAS results using the Cauchy combination test, we found 1,474, 41, 300, 251, and 667 unique genes associated with SmkInit, AgeSmk, CigDay, SmkCes, and DrnkWk, respectively. Across all phenotypes, we identified 2,179 unique genes. Genes identified for AgeSmk were found in an average of 4.3 tissues (SD = 7.6 tissues) including an average of 0.8 brain tissues (SD = 1.6), SmkInit genes were found in an average of 9.5 tissues (SD = 11.0 tissues) including an average of 2.5 brain tissues (SD = 3.4), CigDay genes were found in an average of 9.7 tissues (SD = 11.6 tissues) including an average of 2.6 brain tissues (SD = 3.3), SmkCes genes were found in an average of 8.7 tissues (SD = 10.0 tissues) including an average of 2.2 brain tissues (SD = 3.2), and DrnkWk genes were found in an average of 8.6 tissues (SD = 11.2 tissues) including an average of 2.2 brain tissues (SD = 3.4).

To better understand tissue specificity, we ran parallel and factor analysis of the correlation matrix of TWAS *P*-values across tissues for each gene. For each phenotype parallel analysis[39] suggested that two components explained the majority of the variance in *P*-values. More than half of the variance was explained by the first component for each phenotype (53.7–55.2%), suggesting a general effect across tissues. The second component explained 3.5–3.8% of the variance and was represented primarily by brain tissues, possibly illustrating brain-specific gene expression effects.

We then combined the main TWAS results with pathway information to better identify and prioritize key pathways related to tobacco and alcohol use. A weighted regression approach[40] was used with TWAS *P*-values from each

GTEx tissue to quantify the enrichment of identified genes in the gene pathway domains of molecular function, cellular component, and biological processes (C5 collection from Gene Ontology)[41]. Similar to the main TWAS analysis, we calculated a combined cross-tissue *P*-value for each gene using the Cauchy combination[38] method assigning equal weight to each tissue. We included all results for which a pathway was significantly enriched based on the combined cross-tissue *P*-value in **Supplementary Table 7** (using a Bonferroni *P*-value correction for 10,187 pathways and 5 phenotypes; *P*-value < $9.82 \times 10^{-7}$). Results highlighted important biological pathways for tobacco and alcohol use phenotypes, which were broadly enriched across tissues. For example, acetylcholine-gated channel pathways were enriched in multiple brain tissues for SmkInit, CigDay, and SmkCes. Behavioral response to nicotine pathways, that similarly contain nicotinic receptor (*CHRN*) genes, were significantly enriched across tissues for CigDay and SmkCes. Dopamine receptor signaling and binding pathways, of obvious relevance to neurotransmission, were significantly enriched across tissues for SmkInit, CigDay, and DrnkWk, and alcohol dehydrogenase activity pathways for DrnkWk were enriched in 26 tissues, including 7 (of 13) brain tissues.

## Heritability and genetic correlation

We used univariate LD Score Regression[27] (LDSC) to estimate the heritability of each phenotype and genetic correlations between our five phenotypes for EAS and EUR. For populations with more recent admixture backgrounds (AFR and AMR), existing reference panels of matching ancestry (e.g., 1000G African American) may not be representative of the sample studied here due to differences in admixture proportion and timing which can result in varying LD structures across samples. For this reason, we used covariate-adjusted LD score regression[28] (cov-LDSC) for heritability and genetic correlation estimation in the AFR and AMR populations where we calculated in-sample LD scores and adjusted them by genetic principal components.

For both LDSC and cov-LDSC, we calculated in-sample LD scores using genotypes of TOPMed reference samples. Approximately 20% of the reference samples were randomly selected for each ancestry (6k, 4k, 1k, and 10k for AFR, AMR, EAS, EUR) after removing related individuals (>= 4th degree) using relatedness estimates released by the TOPMed Consortium. LD scores based on random subsamples (greater than 10% of the total sample size) were shown to yield stable estimates of heritability with relatively lower computational burden and well-controlled intercept bias[28]. Within smaller subsamples (e.g., less than 10%), intercepts tended to rise above one even when there was no true confounding, but this bias was better controlled with larger subsampling proportions. Within each population in the TOPMed reference panel, variants were subsetted to HapMap3 with MAF > .05, and variants in high LD regions were removed, in line with the original cov-LDSC recommendations[28]. In EAS and EUR populations, where we used standard LDSC, LD scores were calculated using 1cM window sizes, since LD scores tend to plateau outside of this window range for these populations[27]. The AFR and AMR populations tend to show long-range LD due to recent admixture, thus we used a 20cM window size. When calculating LD scores for AFR and AMR, we accounted for 50 PCs to mitigate a potential downward bias in LDSC heritability estimates due the use of linear mixed-effects models for some contributing summary statistics[28]. We calculated 50 PCs in each TOPMed reference sample where LD was calculated, using LD-pruned (plink1.9 --indep-pairwise 100kb 5 .1) variants with MAF > .05. Based on these methods per ancestry and trait heritability estimates are shown in **Supplementary Table 8**. Cross-trait genetic correlations for each ancestry group are shown in **Supplementary Table 9**.

Using EUR-stratified results for our five smoking and alcohol use phenotypes, we calculated genetic correlations with all UK Biobank phenotypes to inform the relationship that substance use has with a broad array of phenotypes curated in UK Biobank, including diseases, biomarkers, behaviors, and lifestyles. We used pre-calculated summary statistics for UKB phenotype (http://www.nealelab.is/uk-biobank). A total of 4,065 phenotypes were initially considered including 2,328 binary, 1,192 categorical, 274 continuous (rank-based inverse normalized), and 271 ordinal phenotypes. To calculate genetic correlations, we used LDSC with HapMap3 variants and 1000G-based pre-calculated European LD scores.

To further interpret the pattern of genetic correlations, we performed affinity propagation clustering[42], which searches for clusters that maximize net similarity by recursively “passing messages” between data points. It

produces a set of exemplars, which are samples representative of each cluster. We applied “apclusterL” function assuming that our five smoking and drinking phenotypes contain enough information about the cluster structure. After removing phenotypes whose genetic correlations were not properly estimated mostly because of the negative heritabilities, we further excluded UKB phenotypes whose heritability Z-scores were less than three (N = 1,771) as genetic correlations with non-heritable traits are difficult to interpret. We also excluded UKB phenotypes whose genetic correlation estimates were greater than .80 or less than -.80 and those conceptually overlapping with our smoking phenotypes (e.g., “Number of cigarettes currently smoked daily) (N = 21) because they were likely to represent the same constructs with our phenotypes. This resulted in 1,141 phenotypes. We created a similarity matrix (1,141 × 5) using the absolute value of genetic correlations as a similarity metric. We chose to use absolute values since the strength of the relationship between any two given traits will be indexed by the absolute magnitude of the correlation coefficients and the sign of the effect would depend on the way a given phenotype is coded. This clustering algorithm classified each UKB phenotype in one of the five exemplar group (represented by our five smoking/drinking phenotypes). When applying hierarchical clustering at the exemplar-level, Age of Smoking Initiation and Smoking Initiation were grouped together while Cigarettes per Day and Smoking Cessation were grouped. The former (AgeSmk and SmkInit) and latter (CigDay and SmkCes) formed the broad smoking category at a higher-order level, distinct from Drinks per Week. To further interpret the results, we examined phenotypes with relatively high correlations in each exemplar. Visual display of clustering results is presented in **Supplementary Figure 5.** A list of genetic correlations and their assigned clusters is presented in **Supplementary Table 10**.

To evaluate the reliability of our phenotypes across contributing cohorts, we computed leave-one-out genetic correlations for each of the largest five cohorts per phenotype. For example, we computed the genetic correlation between the largest contributing cohort (23andMe) and all other cohorts, leaving out 23andMe for each phenotype (EUR-based results shown in **Supplementary Table 9**). In general, all leave-one-out genetic correlations were high (close to 1) suggesting substantial phenotypic reliability across studies. Due to sampling variability, and because correlation estimates are not bounded in LDSC, some genetic correlations appeared to be greater than one. Leave-one-out genetic correlations within DrnkWk were somewhat smaller in magnitude than for the smoking phenotypes.

When comparing cross-trait genetic correlations within ancestry (e.g., the genetic correlation between SmkInit and CigDay), we noticed an unexpected pattern in which DrnkWk was negatively correlated with CigDay and showed very small genetic correlations with AgeSmk and SmkCes (**Supplementary Table 9**). To better understand these patterns of correlation, we computed leave-one-out cross-trait correlations (restricted to EUR-stratified summary statistics) and found that the genetic correlations for 23andMe differed slightly in magnitude and/or direction than for other cohorts. In other words, the genetic correlation between DrnkWk and CigDay, for example, is -0.16 in the full EUR sample but 0.07 when leaving the 23andMe cohort out (full results in **Supplementary Table 9**). This result could have arisen from confounder or collider bias between DrnkWk and ascertainment, or selection, into 23andMe participation. 23andMe provides a consumer service, and one might expect their consumers to self-select in ways that are different from research studies. That is, if both genetic variation and DrnkWk simultaneously influence consumer behavior to purchase 23andMe services, the associations between those variants and DrnkWk would be affected by collider bias if participation in 23andMe is conditioned on[43].

Relative to a meta-analysis including all studies except 23andMe, DrnkWk in 23andMe alone more strongly positively correlated with indicators of SES, including educational attainment ($r_g$ 0.035 vs. 0.063; from Lee et al.[32]) and income from the UK Biobank ($r_g$ 0.220 vs. 0.084), and negatively correlated with the Townsend index of deprivation from the UK Biobank ($r_g$ -0.101 vs. 0.152). Based on this, we re-estimated genetic correlations, in 23andMe only, between DrnkWk and our smoking phenotypes (AgeSmk, CigDay, SmkCes, and SmkInit) after adjusting for both income and education. After this adjustment, genetic correlations between DrnkWk in 23andMe only and smoking phenotypes became more similar in magnitude to those obtained after excluding 23andMe cohorts. For example, DrnkWk in 23andMe was no longer significantly correlated with AgeSmk ($r_g$ changed from -0.18 to 0.02) and was less strongly correlated with SmkInit ($r_g$ changed from 0.41 to 0.26). The genetic correlation between DrnkWk and CigDay remained negative ($r_g$ = -0.190, SE = 0.024) even after income and education adjustment, though the magnitude decreased slightly.

To explore the issue further, we compared allele frequencies of a U.S. representative sample (AddHealth) with those from our 23andMe DrnkWk results and created summary statistics that indicate selection into 23andMe DrnkWk sample. We found that the selection into 23andMe DrnkWk is genetically correlated with education ($r_g$ = 0.124) and higher income ($r_g$ = 0.137) and, to a lesser degree, with higher alcohol consumption ($r_g$ = 0.06) (**Supplementary Table 11**). This was consistent with previous reports that highly educated individuals are overrepresented in the 23andMe sample[44,45]. However, given the positive, rather than negative, genetic correlation between selection into 23andMe and alcohol consumption, as well as the small magnitude of the association, it appeared that selection into 23andMe was not likely to largely explain the differential patterns of genetic correlations in 23andMe. Indeed, we applied a genomic SEM correction method[46] and found the genetic correlation pattern between DrnkWk-23andMe and other phenotypes did not change appreciably after correcting for selection.

# Polygenic scoring

We assessed how well polygenic risk scores (PRS) for each of our five phenotypes predicted those same phenotypes in an independent prediction sample composed of diverse ancestry individuals: the National Longitudinal Study of Adolescent to Adult Health[47] (Add Health). Add Health used a school-based design to select a nationally representative sample of U.S. adolescents enrolled in grades 7 through 12 during the 1994-1995 school year. Respondents were born between the years of 1974 and 1983 (mean birth year of 1979), and include diverse ancestries of African, Hispanic Admixed, East Asian, and European descent (terminology taken from Add Health). The mean age at the time of assessment used in the current analysis (Wave 4) was 29.1 (SD = 1.8 years).

## Score construction methods

We used LDpred[48], a Bayesian score generation method that takes into account LD information between variants, to construct all polygenic scores. Each reference LD dataset was cohort and ancestry specific, meaning that we used each validation sample as the LD reference panel when there were more than 1,000 individuals left after removing individuals with genetic relatedness coefficients above 0.025. For the East Asian validation dataset in Add Health, because there were less than 1,000 unrelated individuals, we used the East Asian subset of 1000 Genomes as the LD reference sample. For all scores we used an LD radius value of 350 and set the fraction of non-zero effects in the prior to 1. We used ancestry stratified meta-analytic results generated on all studies except for Add Health. Validation datasets were imputed to 1000 Genomes and then ~1.4 million HapMap3 variants (call rate > 98% and MAF > 1%, per ancestry) were extracted for polygenic score creation.

## Score prediction accuracy

For each of the five phenotypes, there were meta-analytic summary statistics based on discovery samples of AFR, AMR, EAS, EUR, and all combined ancestries. The Add Health cohort included individuals of African, Hispanic Admixed, East Asian, and European ancestries, resulting in 20 polygenic scores per phenotype. Combining across phenotypes resulted in 100 polygenic scores. All scores were scaled to have a mean of zero and standard deviation of one.

Prediction accuracy was estimated based on the regression of a given phenotype (SmkInit, AgeSmk, CigDay, SmkCes, and DrnkWk) on the polygenic score along with a set of standard controls, which included age, age$^2$, sex, interaction between age and sex, and the first ten principal components. We first performed this regression without including the PRS. Then, the PRS predictor was added to the regression model and the difference in $R^2$ was calculated. For our quantitative phenotypes, AgeSmk, CigDay, and DrnkWk, the predictive power of the PRS was the change in the $R^2$ between the regression without the PRS to the regression with the PRS. For our two binary phenotypes, SmkInit and SmkCes, we calculated the change in $R^2$ on the liability scale[49] from logistic regressions. 95% confidence intervals around all incremental $R^2$ values were bootstrapped with the replications equal to twice the number of individuals in each model (with no less than 1000 replications). The variable number of bootstrap iterations (2x each model's N) was necessary to obtain stable bias-corrected and accelerated (BCa)

intervals. For the two binary phenotypes, SmkInit and SmkCes, we measured the change in AUC from logistic regressions including only the standard covariates to the regression with the PRS. Bootstrapping was then performed, as implemented in the roc.test() function in the pROC R package, by drawing N (2x the number of individuals in the models) replicates from the data, computing the difference between the AUC of the two ROC curves, standardizing this difference by dividing by the standard deviation of the bootstrap differences, and then comparing to a normal distribution. **Supplementary Table 12** first presents within ancestry results, meaning that we are using PRSs and outcome phenotypic data from the same, matching ancestry (i.e., AgeSmk PRS based on the AFR ancestry stratified meta-analysis to predict age of smoking initiation in the AFR ancestry validation samples). We then present across ancestry validation results in which the discovery sample (GWAS meta-analysis) ancestry does not match the validation sample (Add Health) ancestry.

In addition, we considered whether cross-ancestry scores significantly predicted phenotypes over and above within ancestry scores. In other words, for each ancestry validation sample, we compared a model including base covariates and the within-ancestry PRS to models that incrementally added other ancestry-based PRSs (in alphabetical order). For example, SmkInit validation models with AFR-ancestry validation samples, the predictors of the base model would include the standard covariates plus the AFR-based SmkInit PRS. This base model would then be compared to a model that additionally included the AMR-based SmkInit PRS. The second model would again be compared to additional models that each incrementally add the other ancestry scores (EAS-based SmkInit PRS and EUR-based SmkInit PRS). This resulted in four models that have a nested relationship. The purpose of this analysis was to evaluate whether there were incremental effects of cross-ancestry PRSs over ancestry-matched scores. Full results, including the variance explained in outcome by all four ancestry PRSs (with 95% confidence intervals), are shown in **Supplementary Table 12**. Given the relatively small observed effect sizes and validation sample sizes for some ancestries, we caution that some comparisons may be underpowered to identify differences in the variance explained by polygenic scores between ancestries.

### Bias induced by discovery sample and target sample ancestry mismatch

We estimated expected bias in polygenic prediction methods in non-European ancestries in three ways. First, we compared the distributions of each phenotype PRS, based on a ancestry-stratified meta-analysis, across all other ancestries (i.e., compare the distributions of EUR-based SmkInit polygenic scores in AFR, AMR, EAS, and EUR ancestries). Second, we combined all phenotypic data across ancestry and fit regression models including an interaction term between ancestry group and each PRS, with EUR ancestry as the reference group in order to test whether the PRS performed significantly differently in one ancestry versus another. We also adjusted for all standard covariates as in the validation models. In this way, the interaction terms tested whether the effect of each PRS differed significantly between the ancestry groups. Third, we again combined all phenotypic data across ancestry and fitted regression models including EUR-based PRS only. We compared predicted phenotypes from this model to observed phenotypes within each ancestry group. The predicted vs. observed results were repeated with the inclusion of the base covariates in addition to the EUR-based PRS.

We found that when the ancestry of the PRS discovery sample does not match the ancestry of the target prediction sample, there is a general trend toward pathologizing non-EUR ancestries, particularly when the target sample is composed of individuals of AFR ancestries. In other words, while AFR ancestry individuals in Add Health generally initiate regular smoking at lower rates and at later ages and are more likely to be former smokers compared to other ancestry groups, most polygenic scores, particularly EUR based scores, predict that AFR ancestry individuals have the highest polygenic risk scores for these phenotypes (e.g., EUR based SmkInit scores result in mean PRS values of 1.49 for AFR validation ancestry and -0.55 for EUR validation ancestry). This prediction bias is readily removed by correcting for genetic principal components.

## Contributions and acknowledgements

### Detailed author contributions

Dajiang J. Liu and Scott Vrieze designed, led, and oversaw the study.

Gretchen Saunders and Xingyan Wang were the study's lead analysts, responsible for quality control, meta-analyses, and a wide variety of other tasks; they were assisted by Dajiang J. Liu, Scott Vrieze, Fang Chen, Seon-Kyeong Jang, Mengzhen Liu, and Chen Wang.

Phenotype definitions were developed by Laura J. Bierut, Marilyn C. Cornelis, David A. Hinds, Jaakko Kaprio, Eric Jorgenson, Dajiang J. Liu, Matt McGue, Marcus R. Munafo, Scott Vrieze, and Luisa Zuccolo.

Software development and implementation were carried out by Xingyan Wang, Dajiang J. Liu, Fang Chen, and Chen Wang.

Multi-ancestry meta-analyses were performed by Xingyan Wang.
Ancestry-stratified meta-analyses were performed by Gretchen Saunders and Mengzhen Liu.
Conditional analyses were performed by Xingyan Wang and Gretchen Saunders.
Fine-mapping and allelic heterogeneity analyses were performed by Xingyan Wang and Gretchen Saunders.
Replicability analyses were performed by Chen Wang, Seon-Kyeong Jang, and Gretchen Saunders.
Multi-ancestry TWAS and enrichment analyses were performed by Fang Chen.
Heritability and genetic correlation analyses were performed by Seon-Kyeong Jang.
Polygenic scoring analyses were performed by Gretchen Saunders.
Functional enrichment of fine-mapped variants was performed by Seon-Kyeong Jang.

Bioinformatics and biological insights analyses were performed by Fang Chen (TESLA), Gretchen Saunders (GWAS catalogue lookup; manual review of implicated genes), and Scott Vrieze (GWAS catalogue lookup; manual review of implicated genes). In interpreting results, they were assisted by Jerry A. Stitzel. The majority of figures were created by Mengzhen Liu and Gretchen Saunders.

Mengzhen Liu and Scott Vrieze helped with coordinating among the participating cohorts. Marissa A. Ehringer and Matthew C. Keller helped with data access. Gretchen Saunders managed and coordinated all authorship and acknowledgement details for the study.

Gretchen Saunders wrote the manuscript draft with significant components from Xingyan Wang, Seon-Kyeong Jang, Fang Chen, and Chen Wang, helpful suggestions and additions from Mengzhen Liu, Chiara Batini, Andrew Bergen, Laura Bierut, Sean David, Sarah Gagliano Talium, Dana Hancock, Marcus Munafò, Jerry A. Stitzel, and Thorgeir Thorgeirsson, and oversight from Dajiang Liu and Scott Vrieze.

Chiara Batini, Andrew Bergen, Laura Bierut, Sean David, Sarah Gagliano Talium, Dana Hancock, Marcus Munafò, and Thorgeir Thorgeirsson provided particularly helpful advice and feedback on various aspects of the study design and the manuscript.

## Cohort-level acknowledgements

**TOPMed (Trans-Omics for Precision Medicine) –** WGS for the TOPMed program was supported by the National Heart, Lung and Blood Institute (NHLBI). Specific funding sources and acknowledgements for each study are provided below. Centralized read mapping and genotype calling, along with variant quality metrics and filtering were provided by the TOPMed Informatics Research Center (3R01HL-117626-02S1; contract HHSN268201800002I). Phenotype harmonization, data management, sample-identity quality control and general study coordination were provided by the TOPMed Data Coordinating Center (R01HL-120393; U01HL-120393; contract HHSN268201800001I). We thank the studies and participants who provided biological samples and data for TOPMed. The views expressed in this manuscript are those of the authors and do not necessarily represent the views of the National Heart, Lung, and Blood Institute; the National Institutes of Health; or the US Department of Health and Human Services.

**23andMe, Inc.** – 23andMe participants provided informed consent and participated in the research online, under a protocol approved by the external AAHRPP-accredited IRB, Ethical & Independent Review Services (E&I Review). Participants were included in the analysis on the basis of consent status as checked at

the time data analyses were initiated. The name of the IRB at the time of the approval was Ethical & Independent Review Services. Ethical & Independent Review Services was recently acquired, and its new name as of July 2022 is Salus IRB (https://www.versiticlinicaltrials.org/salusirb). We would like to thank the research participants and employees of 23andMe for making this work possible. The full GWAS summary statistics for the 23andMe datasets will be made available to qualified researchers under an agreement with 23andMe that protects the privacy of the 23andMe participants. Please contact apply.research@23andme.com for more information and to apply to access the data.

The following members of the 23andMe Research Team contributed to this study: Stella Aslibekyan, Adam Auton, Elizabeth Babalola, Robert K. Bell, Jessica Bielenberg, Katarzyna Bryc, Emily Bullis, Daniella Coker, Gabriel Cuellar Partida, Devika Dhamija, Sayantan Das, Sarah L. Elson, Teresa Filshtein, Kipper Fletez-Brant, Pierre Fontanillas, Will Freyman, Pooja M. Gandhi, Karl Heilbron, Barry Hicks, David A. Hinds, Ethan M. Jewett, Yunxuan Jiang, Katelyn Kukar, Keng-Han Lin, Maya Lowe, Jey C. McCreight, Matthew H. McIntyre, Steven J. Micheletti, Meghan E. Moreno, Joanna L. Mountain, Priyanka Nandakumar, Elizabeth S. Noblin, Jared O'Connell, Aaron A. Petrakovitz, G. David Poznik, Morgan Schumacher, Anjali J. Shastri, Janie F. Shelton, Jingchunzi Shi, Vinh Tran, Joyce Y. Tung, Xin Wang, Wei Wang, Catherine H. Weldon, Peter Wilton, Alejandro Hernandez, Corinna Wong, Christophe Toukam Tchakouté.

**AACAC (African American Coronary Artery Calcification project of the MESA Family Study) –** MESA Family is conducted and supported by the National Heart, Lung, and Blood Institute (NHLBI) in collaboration with MESA investigators. Support is provided by grants and contracts R01HL071051, R01HL071205, R01HL071250, R01HL071251, R01HL071258, R01HL071259, by the National Center for Research Resources, Grant UL1RR033176. The Diabetes Heart Study (DHS) was supported by R01 HL92301, R01 HL67348, R01 NS058700, R01 AR48797, R01 DK071891, R01 AG058921, the General Clinical Research Center of the Wake Forest University School of Medicine (M01 RR07122, F32 HL085989), the American Diabetes Association, and a pilot grant from the Claude Pepper Older Americans Independence Center of Wake Forest University Health Sciences (P60 AG10484). The datasets used for the analyses described in this manuscript were obtained from dbGaP at http://www.ncbi.nlm.nih.gov/gap through dbGaP accession number phs001412.

**Add Health (National Longitudinal Study of Adolescent to Adult Health)** – This research uses data from Add Health, funded by grant P01 HD31921 (Harris) from the *Eunice Kennedy Shriver* National Institute of Child Health and Human Development (NICHD), with cooperative funding from 23 other federal agencies and foundations. Add Health is currently directed by Robert A. Hummer and funded by the National Institute on Aging cooperative agreements U01 AG071448 (Hummer) and U01AG071450 (Aiello and Hummer) at the University of North Carolina at Chapel Hill. Add Health was designed by J. Richard Udry, Peter S. Bearman, and Kathleen Mullan Harris at the University of North Carolina at Chapel Hill. GWAS data were funded by Eunice Kennedy Shriver National Institute of Child Health and Human Development (NICHD) Grants R01 HD073342 (Harris) and R01 HD060726 (Harris, Boardman, and McQueen). Investigators thank the staff and participants of the Add Health Study for their important contributions.

**ALSPAC (Avon Longitudinal Study of Parents and Children)** – We are extremely grateful to all the families who took part in this study, the midwives for their help in recruiting them, and the whole ALSPAC team, which includes interviewers, computer and laboratory technicians, clerical workers, research scientists, volunteers, managers, receptionists and nurses. The UK Medical Research Council and Wellcome (Grant ref: 217065/Z/19/Z) and the University of Bristol provide core support for ALSPAC. This research was performed in the UK Medical Research Council Integrative Epidemiology Unit (grant numbers MC_UU_00011/6, MC_UU_00011/7) and also supported by the National Institute for Health Research (NIHR) Bristol Biomedical Research Centre at University Hospitals Bristol NHS Foundation Trust and the University of Bristol. LZ is supported by a UK Medical Research Council fellowship (grant number G0902144). A comprehensive list of grants funding is available on the ALSPAC website (http://www.bristol.ac.uk/alspac/external/documents/grant-acknowledgements.pdf).

Descriptions of the ALSPAC cohort can be found in the two following articles: (1) Boyd A, Golding J, Macleod J, Lawlor DA, Fraser A, Henderson J, Molloy L, Ness A, Ring S, Davey Smith G. Cohort Profile: The ‘Children of the 90s’; the index offspring of The Avon Longitudinal Study of Parents and Children (ALSPAC). *International Journal of Epidemiology* 2013; 42:111-127; (2) Fraser A, Macdonald-Wallis C, Tilling K, Boyd A, Golding J, Davey Smith G, Henderson J, Macleod J, Molloy L, Ness A, Ring S, Nelson SM, Lawlor DA. Cohort Profile: The Avon Longitudinal Study of Parents and Children: ALSPAC mothers cohort. *International Journal of*

*Epidemiology* 2013; 42:97- 110. Study data for individuals 22 years and older were collected and managed using REDCap electronic data capture tools hosted at the University of Bristol. REDCap (Research Electronic Data Capture) is a secure, web-based application designed to support data capture for research studies. The tool is described in detail in the following article: Paul A. Harris, Robert Taylor, Robert Thielke, Jonathon Payne, Nathaniel Gonzalez, Jose G. Conde, Research electronic data capture (REDCap) – A metadata-driven methodology and workflow process for providing translational research informatics support, *Journal of Biomedical Informatics* 2009; 42(2):377-381.

Please note that the ALSPAC study website contains details of all the data that is available through a fully searchable data dictionary available here: http://www.bris.ac.uk/alspac/researchers/data-access/data-dictionary/. Ethical approval for the study was obtained from the ALSPAC Ethics and Law Committee and the Local Research Ethics Committees. Details on the ethics committee/institutional review board that approved aspects of the study can be found here: http://www.bristol.ac.uk/alspac/researchers/research-ethics/. For more information about this dataset, see http://www.bristol.ac.uk/alspac/.

**AMISH (Genetics of Cardiometabolic Health in the Amish)** – The Amish studies upon which these data are based were supported by NIH grants R01 AG18728, U01 HL072515, R01 HL088119, and R01 HL121007. The Amish Research Program gratefully acknowledges the contributions of the participants and of the study staff. Robert M. Reed was supported by the Flight Attendants Medical Research Institute (FAMRI). The datasets used for the analyses described in this manuscript were obtained from dbGaP at http://www.ncbi.nlm.nih.gov/gap through dbGaP accession number phs000956.

**ARIC (Atherosclerosis Risk in Communities)** – The Atherosclerosis Risk in Communities (ARIC) study has been funded in whole or in part with Federal funds from the National Heart, Lung, and Blood Institute, National Institutes of Health, Department of Health and Human Services, under contract numbers HHSN268201700001I, HHSN268201700002I, HHSN268201700003I, HHSN268201700004I, HHSN268201700005I. The authors thank the staff and participants of the ARIC study for their important contributions. The datasets used for the analyses described in this manuscript were obtained from dbGaP at http://www.ncbi.nlm.nih.gov/gap through dbGaP accession number phs001211.

**BAGS (Barbados Asthma Genetics Study)** – The authors wish to thank the families in Barbados and volunteers participating in BAGS. We are grateful to Drs. Harold Watson and Clive Landis and Pissamai Maul, Trevor Maul, and Desiree Walcott for their contributions in the field and support at the Chronic Disease Research Centre. Funding for BAGS was provided by National Institutes of Health (NIH) R01HL104608, R01HL087699, HL104608 S1, R01AI132476, and R01AI114555. The datasets used for the analyses described in this manuscript were obtained from dbGaP at http://www.ncbi.nlm.nih.gov/gap through dbGaP accession number phs001143.

**BEAGESS (The Barrett's and Esophageal Adenocarcinoma Genetic Susceptibility Study) –** This study made use of data generated by investigators in the BEACON consortium through a grant funded by the US National Institutes of Health (NIH) (RO1CA136725) to Thomas L. Vaughan and David C. Whiteman (multiple PIs). In support of this work, T.L.V. was also supported by NIH grant KO5CA124911 and D.C.W. by a Future Fellowship grant FT0990987 from the Australia Research Council. Additional collaborators, sources of support and origin of the data and biospecimens are listed in the following publication: Levine DM, Ek WE, Zhang R, Liu X, Onstad L, Sather C, et al. A genome-wide association study identifies new susceptibility loci for esophageal adenocarcinoma and Barrett's esophagus. Nat Genet. 2013 Dec;45(12):1487–93. The dataset used for the analyses described in this manuscript was obtained from dbGaP at http://www.ncbi.nlm.nih.gov/gap through dbGaP accession number phs000869.

**Biobank Japan** – The Biobank Japan (BBJ) Project was established in 2003 with the aim of the implementation of personalized medicine as a leading project of Ministry of Education, Culture, Sports, Science and Technology (MEXT). In collaboration with twelve cooperating institutes, the BBJ has recruited a total of 200,000 people, suffering from at least one of the 47 target common diseases, in the first phase (5-year period) . BBJ has collected biospecimens including DNA and serum as well as various clinical and lifestyle information through interview or medical records by using standardized questionnaire. All participants gave written informed consent to this project and this study was approved by ethical committees of RIKEN and participating institutes. For more information about this study, please see https://biobankjp.org/english/plan/summary.html.

BBJ was supported by the Tailor-Made Medical Treatment Program (the BioBank Japan Project) of the Ministry of Education, Culture, Sports, Science, and Technology (MEXT), and the Japan Agency for Medical Research and Development (AMED) (grant ID: JP17km0305002). We acknowledge all patients who participated

in the study. We thank the staff of the BBJ for their collecting and managing of clinical information and samples. We also thank the contributions of the Tohoku Medical Megabank Project, the Japan Public Health Center-based Prospective (JPHC) Study, the Japan Multi-Institutional Collaborative Cohort (J-MICC) Study.

Y.O. was supported by JSPS KAKENHI (22H00476), and AMED (JP21km0405211, JP21ek0109413, JP21ek0410075, JP21gm4010006, and JP21km0405217), JST Moonshot R&D (JPMJMS2021, JPMJMS2024), Takeda Science Foundation, Bioinformatics Initiative of Osaka University Graduate School of Medicine, and Center for Infectious Disease Education and Research (CiDER), Osaka University, and Institute for Open and Transdisciplinary Research Initiatives, Osaka University.

**BLTS (Brisbane Longitudinal Twin Study)** – The Brisbane Longitudinal Study acknowledges funding from the Australian National Health and Medical Research Council grants 1031119 and 1049911. For more information about this study, contact Nathan A. Gillespie (nathan.gillespie@vcuhealth.org).

**EOCOPD (Boston Early-Onset COPD Study) –** The Boston Early-Onset COPD Study was supported by the following NIH grants: R01 HL075478, R01 HL089856, and R01 HL113264. The dataset used for the analyses described in this manuscript was obtained from dbGaP at http://www.ncbi.nlm.nih.gov/gap through dbGaP accession number phs000946.

**CADD (Center on Antisocial Drug Dependence)** – The Center on Antisocial Drug Dependence (CADD) data were funded by grants from the National Institute on Drug Abuse (P60 DA011015, R01 DA012845, R01 DA035804). The Genetics of Adolescent Drug Dependence (GADD) acknowledges the contributions of the participants and study staff. For more information about this study, contact John K. Hewitt (john.hewitt@colorado.edu).

**CFS (Cleveland Family Study) –** The Cleveland Family Study has been supported by National Institutes of Health grants [R01-HL046380, KL2-RR024990, R35-HL135818, and R01-HL113338]. Brian E. Cade was funded by NIH grants K01HL135405 and R03HL154284. The dataset used for the analyses described in this manuscript was obtained from dbGaP at http://www.ncbi.nlm.nih.gov/gap through dbGaP accession number phs000954.

**China Kadoorie Biobank –** The China Kadoorie Biobank (CKB) baseline survey and the first re-survey were supported by the Kadoorie Charitable Foundation in Hong Kong. Long-term follow-up was supported by the Wellcome Trust (212946/Z/18/Z, 202922/Z/16/Z, 104085/Z/14/Z, 088158/Z/09/Z), the National Key Research and Development Program of China (2016YFC0900500, 2016YFC0900501, 2016YFC0900504, 2016YFC1303904), and the National Natural Science Foundation of China (91843302). DNA extraction and genotyping was funded by GlaxoSmithKline and the UK Medical Research Council (MC-PC-13049, MC-PC-14135). The project is supported by core funding from the UK Medical Research Council (MC_UU_00017/1,MC_UU_12026/2, MC_U137686851), Cancer Research UK (C16077/A29186; C500/A16896), and the British Heart Foundation (CH/1996001/9454) to the Clinical Trial Service Unit and Epidemiological Studies Unit and to the MRC Population Health Research Unit at Oxford University. CKB gratefully acknowledges the participants in the study, the members of the survey teams in each of the 10 regional centres, and the project development and management teams based at Beijing, Oxford and the 10 regional centres.

**CHS (Cardiovascular Health Study)** – This CHS research was supported by NHLBI contracts HHSN268201200036C, HHSN268200800007C, HHSN268201800001C, N01HC55222, N01HC85079, N01HC85080, N01HC85081, N01HC85082, N01HC85083, N01HC85086, 75N92021D00006; and NHLBI grants U01HL080295, R01HL085251, R01HL087652, R01HL105756, R01HL103612, R01HL120393, and U01HL130114 with additional contribution from the National Institute of Neurological Disorders and Stroke (NINDS). Additional support was provided through R01AG023629 from the National Institute on Aging (NIA). A full list of principal CHS investigators and institutions can be found at CHS-NHLBI.org.

The provision of genotyping data was supported in part by the National Center for Advancing Translational Sciences, CTSI grant UL1TR001881, and the National Institute of Diabetes and Digestive and Kidney Disease Diabetes Research Center (DRC) grant DK063491 to the Southern California Diabetes Endocrinology Research Center. The content is solely the responsibility of the authors and does not necessarily represent the official views of the National Institutes of Health.The dataset used for the analyses described in this manuscript was obtained from dbGaP at http://www.ncbi.nlm.nih.gov/gap through dbGaP accession number phs001368.

**COGEND (Collaborative Genetic Study of Nicotine Dependence)** – This research was supported by P01 CA089392, U01 HG004422, and R01 DA036583. Funding support for genotyping which was performed at

the Johns Hopkins University Center for Inherited Disease Research was provided by the NIH "Genome-wide Association Studies in the Genes and Environment Initiative" (U01HG004438) and the NIH contract "High throughput genotyping for studying the genetic contributions to human disease" (HHSN268200782096C). We also acknowledge funding from R01 DA026911. For more information about this study, contact Laura J. Bierut (laura@wustl.edu).

**COPDGene (Genetic Epidemiology of COPD)** – COPDGene was supported by U01 HL089897 and U01 HL089856 from the National Heart, Lung, and Blood Institute. The content is solely the responsibility of the authors and does not necessarily represent the official views of the National Heart, Lung, and Blood Institute or the National Institutes of Health. COPDGene is also supported by the COPD Foundation through contributions made to an Industry Advisory Board that has included AstraZeneca, Bayer Pharmaceuticals, Boehringer-Ingelheim, Genentech, GlaxoSmithKline, Novartis, Pfizer, and Sunovion. A full listing of COPDGene investigators can be found at: http://www.copdgene.org/directory. The dataset used for the analyses described in this manuscript was obtained from dbGaP at http://www.ncbi.nlm.nih.gov/gap through dbGaP accession number phs000951.

**CRA (The Genetic Epidemiology of Asthma in Costa Rica)** – This study was supported by NHLBI grant P01 HL132825. We wish to acknowledge the investigators at the Channing Division of Network Medicine at Brigham and Women's Hospital, the investigators at the Hospital Nacional de Niños in San José, Costa Rica and the study subjects and their extended family members who contributed samples and genotypes to the study, and the NIH/NHLBI for its support in making this project possible. The dataset used for the analyses described in this manuscript was obtained from dbGaP at http://www.ncbi.nlm.nih.gov/gap through dbGaP accession number phs000988.

**deCODE (deCODE Genetics/AMGEN, Inc.)** – The authors are thankful to the Icelandic participants and staff at the Patient Recruitment Center. The work at deCODE genetics / Amgen was supported in part by the National Institute of Drug Abuse (NIDA grants, R01-DA017932 and R01-DA034076). For more information about this study, email info@decode.is.

**ECLIPSE (Evaluation of COPD Longitudinally to Identify Predictive Surrogate Endpoints)** – The Evaluation of COPD Longitudinally to Identify Predictive Surrogate End-points (ECLIPSE) study (SCO104960, NCT00292552) was sponsored by GSK. The dataset used for the analyses described in this manuscript was obtained from dbGaP at http://www.ncbi.nlm.nih.gov/gap through dbGaP accession number phs001472.

**EGCUT (Estonian Genome Center)** – The EGCUT studies were supported by the European Union through the European Regional Development Fund (Project No. 2014-2020.4.01.15-0012), by the Estonian Research Council grant PUT (PRG687), and by the Estonian Research Council grant PUT (PRG1291). We acknowledge the Estonian Biobank research team. Data analyses were carried out in part in the High-Performance Computing Center of University of Tartu.

**eMERGE (Electronic Medical Records and Genomics) –** Samples and associated genotype and phenotype data used in this study were provided by the Mayo Clinic. Funding support for the Mayo Clinic was provided through a cooperative agreement with the National Human Genome Research Institute (NHGRI), Grant number: U01HG004599; and by grant HL75794 from the National Heart Lung and Blood Institute (NHLBI). Funding support for genotyping, which was performed at The Broad Institute, was provided by the NIH (U01HG004424). Assistance with phenotype harmonization and genotype data cleaning was provided by the eMERGE Administrative Coordinating Center (U01HG004603) and the National Center for Biotechnology Information (NCBI). The datasets used for analyses described in this manuscript were obtained from dbGaP at http://www.ncbi.nlm.nih.gov/gap through dbGaP accession number phs000203.

Funding support for the Personalized Medicine Research Project (PMRP) was provided through a cooperative agreement (U01HG004608) with the National Human Genome Research Institute (NHGRI), with additional funding from the National Institute for General Medical Sciences (NIGMS) The samples used for PMRP analyses were obtained with funding from Marshfield Clinic, Health Resources Service Administration Office of Rural Health Policy grant number D1A RH00025, and Wisconsin Department of Commerce Technology Development Fund contract number TDF FYO10718. Funding support for genotyping, which was performed at Johns Hopkins University, was provided by the NIH (U01HG004438). Assistance with phenotype harmonization and genotype data cleaning was provided by the eMERGE Administrative Coordinating Center (U01HG004603) and the National Center for Biotechnology Information (NCBI). The datasets used for the analyses described in this manuscript were obtained from dbGaP at http://www.ncbi.nlm.nih.gov/gap through dbGaP accession number phs000170.

**FinnTwin, FINRISK, & NAG-FIN (Finnish Twin Cohort)** – The Finnish Twin Cohort/Nicotine Addiction Genetics-Finland study was supported by Academy of Finland Center of Excellence in Complex Disease Genetics (grant numbers: 213506, 129680, 312073) , the Academy of Finland (grants 100499, 205585, 118555, 141054, 265240, 263278, 264146 to J. Kaprio), Sigrid Juselius Foundation (to J. Kaprio) , Global Research Awards for Nicotine Dependence (GRAND) , ENGAGE – European Network for Genetic and Genomic Epidemiology, FP7-HEALTH-F4-2007, grant agreement number 201413, DA12854 to P.A.F. Madden, and AA-12502, AA-00145, and AA-09203 to R.J. Rose, AA15416 and K02AA018755 to D.M. Dick. We thank all the participants for taking part in the studies and the research staff, who have been involved in data collection and genotyping. For more information about this study, contact Jaakko Kaprio (jaakko.kaprio@helsinki.fi).

**FHS (Framingham Heart Study)** – The Framingham Heart Study is conducted and supported by the National Heart, Lung, and Blood Institute (NHLBI) in collaboration with Boston University (NIH grants 75N92019D00031; 2U54HL120163; R01HL092577). The FHS gratefully acknowledges the contributions of the participants and staff of the study. This manuscript was not prepared in collaboration with investigators of the Framingham Heart Study and does not necessarily reflect the opinions or views of the Framingham Heart Study, Boston University, or NHLBI. Funding for SHARe Affymetrix genotyping was provided by NHLBI Contract N02-HL64278. SHARe Illumina genotyping was provided under an agreement between Illumina and Boston University. Funding for Affymetrix genotyping of the FHS Omni cohorts was provided by Intramural NHLBI funds from Andrew D. Johnson and Christopher J. O'Donnell. Chunyu Liu was funded by NIH grant R01AA028263. The datasets used for the analyses described in this manuscript were obtained from dbGaP at http://www.ncbi.nlm.nih.gov/gap through dbGaP accession number phs000974.

**GeneSTAR (Genetic Studies of Atherosclerosis Risk) –** GeneSTAR was supported by the National Institutes of Health/National Heart, Lung, and Blood Institute (U01 HL72518, HL087698, HL112064) and by a grant from the National Institutes of Health/National Center for Research Resources (M01-RR000052) to the Johns Hopkins General Clinical Research Center. GeneSTAR thanks our participants and staff for their valuable contributions. The datasets used for the analyses described in this manuscript were obtained from dbGaP at http://www.ncbi.nlm.nih.gov/gap through dbGaP accession number phs001218.

**GENOA (Genetic Epidemiology Network of Arteriopathy) –** Support for GENOA was provided by the National Heart, Lung and Blood Institute (U01 HL054457, U01 HL054464, U01 HL054481, R01 HL087660, and R01 HL119443) of the National Institutes of Health. We would like to thank the families that participated in the GENOA study. The datasets used for the analyses described in this manuscript were obtained from dbGaP at http://www.ncbi.nlm.nih.gov/gap through dbGaP accession number phs001345.

**GENSalt (Genetic Epidemiology Network of Salt Sensitivity) –** GenSalt was supported by research grants (U01HL072507, R01HL087263, and R01HL090682) from the NHLBI and partially supported by the National Institute of General Medical Sciences of the NIH under Award Number P20GM109036 and the Collins C. Diboll Private Foundation, New Orleans, LA. The datasets used for the analyses described in this manuscript were obtained from dbGaP at http://www.ncbi.nlm.nih.gov/gap through dbGaP accession number phs001217.

**GERA (Genetic Epidemiology Research in Adult Health and Aging) –** We are grateful to the Kaiser Permanente Northern California members who have generously agreed to participate in the Kaiser Permanente Research Program on Genes, Environment, and Health. Genotyping of the GERA cohort was funded by a grant from the National Institute on Aging, National Institute of Mental Health, and National Institute of Health Common Fund [RC2 AG036607]. Support for GERA participant enrollment, survey completion, and biospecimen collection for the Research Program on Genes, Environment and Health was provided by the Robert Wood Johnson Foundation, the Wayne and Gladys Valley Foundation, the Ellison Medical Foundation, and Kaiser Permanente Community Benefit Programs.

**GfG (Genes for Good) –** The Genes for Good study is funded through discretionary funds, provided to Dr. Gonçalo Abecasis by the University of Michigan. GfG gratefully acknowledges our participants' help in developing GfG as a data resource for understanding the link between genes and common heritable traits. The authors sincerely thank all study participants for their time and dedication, as well as the hard-working Genes for Good administrative staff and colleagues at the UM DNA Sequencing Core. For more information about this study, see https://genesforgood.sph.umich.edu/ or contact the study directly at genesforgood@umich.edu.

**GOLDN (Genetics of Lipid Lowering Drugs and Diet Network) –** GOLDN biospecimens, baseline phenotype data, and intervention phenotype data were collected with funding from National Heart, Lung and Blood Institute (NHLBI) grant U01 HL072524. Whole-genome sequencing in GOLDN was funded by NHLBI grant R01 HL104135-04S1. The datasets used for the analyses described in this manuscript were obtained from

dbGaP at http://www.ncbi.nlm.nih.gov/gap through dbGaP accession number phs001359.

**NHS, NHS2, and HPFS (Nurses' Health Study, Nurses' Health Study II, and Health Professionals' Follow-up Study)** — The contributions from the Nurses' Health Study, Nurses' Health Study II, and Health Professionals' Follow-up Study were supported by the National Institute of Health grants P01CA87969, P01CA055075, P01DK070756, U01HG004728, UM1CA186107, UM1CA176726, UM1CA167552, R01CA49449, R01CA50385, R01CA131332, R01CA67262, R01HL034594, R01HL088521, R01HL116854, R01HL35464, R01EY015473, R01EY022305, P30EY014104, R03DC013373, and R03CA165131. We thank all participants of the NHS, NHS II and HPFS for their continued contributions to research. For information about these studies, contact Peter Kraft pkraft@hsph.harvard.edu) or Marilyn C. Cornelis (marilyn.cornelis@northwestern.edu).

**HCHS SOL (Hispanic Community Health Study - Study of Latinos) –** The Hispanic Community Health Study/Study of Latinos is a collaborative study supported by contracts from the National Heart, Lung, and Blood Institute (NHLBI) to the University of North Carolina (HHSN268201300001I / N01-HC-65233), University of Miami (HHSN268201300004I / N01-HC-65234), Albert Einstein College of Medicine (HHSN268201300002I / N01-HC-65235), University of Illinois at Chicago (HHSN268201300003I / N01-HC-65236 Northwestern Univ), and San Diego State University (HHSN268201300005I / N01-HC-65237). The following Institutes/Centers/Offices have contributed to the HCHS/SOL through a transfer of funds to the NHLBI: National Institute on Minority Health and Health Disparities,National Institute on Deafness and Other Communication Disorders, National Institute of Dental and Craniofacial Research, National Institute of Diabetes and Digestive and Kidney Diseases, National Institute of Neurological Disorders and Stroke, NIH Institution-Office of Dietary Supplements. The datasets used for the analyses described in this manuscript were obtained from dbGaP at http://www.ncbi.nlm.nih.gov/gap through dbGaP accession number phs001395.

**HRS (Health and Retirement Study)** — HRS is supported by the National Institute on Aging (NIA U01AG009740). The genotyping was funded separately by the National Institute on Aging (RC2 AG036495, RC4 AG039029). Our genotyping was conducted by the NIH Center for Inherited Disease Research (CIDR) at Johns Hopkins University. Genotyping quality control and final preparation of the data were performed by the University of Michigan School of Public Health. See the HRS website (http://hrsonline.isr.umich.edu/gwas) for details.

**HUNT (The Nord-Trøndelag Health Study)** — The Trøndelag Health Study (HUNT) is a collaboration between HUNT Research Centre (Faculty of Medicine and Health Sciences, Norwegian University of Science and Technology NTNU), Trøndelag County Council, Central Norway Regional Health Authority, and the Norwegian Institute of Public Health. The genotyping was financed by the National Institute of health (NIH), University of Michigan, The Norwegian Research council, and Central Norway Regional Health Authority and the Faculty of Medicine and Health Sciences, Norwegian University of Science and Technology (NTNU). The genotype quality control and imputation has been conducted by the K.G. Jebsen center for genetic epidemiology, Department of public health and nursing, Faculty of medicine and health sciences, Norwegian University of Science and Technology (NTNU).

**HVH (Heart and Vascular Health Study)** – The Heart and Vascular Health Study was supported by grants HL068986, HL085251, HL095080, and HL073410 from the National Heart, Lung, and Blood Institute. The datasets used for the analyses described in this manuscript were obtained from dbGaP at http://www.ncbi.nlm.nih.gov/gap through dbGaP accession number phs000993.

**HyperGEN (Hypertension Genetic Epidemiology Network)** – The Hypertension Genetic Epidemiology Network (HyperGEN) Study is part of the National Heart, Lung, and Blood Institute (NHLBI) Family Blood Pressure Program; collection of the data represented here was supported by grants U01 HL054472, U01 HL054473, U01 HL054495, and U01 HL054509. The HyperGEN: Genetics of Left Ventricular Hypertrophy Study was supported by NHLBI grant R01 HL055673 with whole-genome sequencing made possible by supplement -18S1. The datasets used for the analyses described in this manuscript were obtained from dbGaP at http://www.ncbi.nlm.nih.gov/gap through dbGaP accession number phs001293.

**IPF (Familial and Sporadic Idiopathic Pulmonary Fibrosis)** – IPF is supported by the following grants: W81XWH-17-1-0597, UG3/UH3-HL151865, P01-HL0928701, R01-HL097163, X01-HL134585. The datasets used for the analyses described in this manuscript were obtained from dbGaP at http://www.ncbi.nlm.nih.gov/gap through dbGaP accession number phs001607.

**JHS (Jackson Heart Study) –** The Jackson Heart Study (JHS) is supported and conducted in collaboration with Jackson State University (HHSN268201800013I), Tougaloo College (HHSN268201800014I), the Mississippi State Department of Health (HHSN268201800015I) and the University of Mississippi Medical

Center (HHSN268201800010I, HHSN268201800011I and HHSN268201800012I) contracts from the National Heart, Lung, and Blood Institute (NHLBI) and the National Institute on Minority Health and Health Disparities (NIMHD). The authors also wish to thank the staffs and participants of the JHS. The datasets used for the analyses described in this manuscript were obtained from dbGaP at http://www.ncbi.nlm.nih.gov/gap through dbGaP accession number phs000964.

**MCTFR (Minnesota Center for Twin and Family Research) –** MCTFR was supported in part by USPHS Grants from the National Institute on Alcohol Abuse and Alcoholism (R01 AA09367 and R01 AA11886) and from the National Institute on Drug Abuse (R01 DA05147, R01 DA13240, R01DA042755, U01DA046413, R21DA046188, R01DA037904, R01HG008983, and U01DA024417). GWAS and phenotypic data for MCTFR subjects who provided consent to place their data in a public repository are deposited into the database of Genotypes and Phenotypes (dbGaP, www.ncbi.nlm.nih.gov/gap) under phs000620. For further information, please contact Scott Vrieze (vrieze@umn.edu). Scott Vrieze is funded by NIH grants R56 HG011035, R01 DA044283, R01 DA037904, and through the Minnesota Supercomputing Institute.

**MESA (Multi-Ethnic Study of Atherosclerosis)** — Molecular data for the Trans-Omics in Precision Medicine (TOPMed) program was supported by the National Heart, Lung and Blood Institute (NHLBI). Multi-Ethnic Study of Atherosclerosis (MESA) (phs001416.v1.p1) was performed at the Broad Institute of MIT and Harvard (3U54HG003067-13S1). Centralized read mapping and genotype calling, along with variant quality metrics and filtering were provided by the TOPMed Informatics Research Center (3R01HL-117626-02S1, contract HHSN268201800002I) (Broad RNA Seq, Proteomics HHSN268201600034I, UW RNA Seq HHSN268201600032I, USC DNA Methylation HHSN268201600034I, Broad Metabolomics HHSN268201600038I). Phenotype harmonization, data management, sample-identity QC, and general study coordination, were provided by the TOPMed Data Coordinating Center (3R01HL-120393; U01HL-120393; contract HHSN268180001I). The MESA projects are conducted and supported by the National Heart, Lung, and Blood Institute (NHLBI) in collaboration with MESA investigators. Support for the Multi-Ethnic Study of Atherosclerosis (MESA) projects are conducted and supported by the National Heart, Lung, and Blood Institute (NHLBI) in collaboration with MESA investigators. Support for MESA is provided by contracts 75N92020D00001, HHSN268201500003I, N01-HC-95159, 75N92020D00005, N01-HC-95160, 75N92020D00002, N01-HC-95161, 75N92020D00003, N01-HC-95162, 75N92020D00006, N01-HC-95163, 75N92020D00004, N01-HC-95164, 75N92020D00007, N01-HC-95165, N01-HC-95166, N01-HC-95167, N01-HC-95168, N01-HC-95169, UL1-TR-000040, UL1-TR-001079, UL1-TR-001420, UL1TR001881, DK063491, and R01HL105756. The authors thank the other investigators, the staff, and the participants of the MESA study for their valuable contributions. A fill list of participating MESA investigators and institutes can be found at http://www.mesa-nhlbi.org. The datasets used for the analyses described in this manuscript were obtained from dbGaP at http://www.ncbi.nlm.nih.gov/gap through dbGaP accession number phs001416.

**METSIM (Metabolic Syndrome in Men)** — The METSIM study was funded by the Academy of Finland (grant no.77299 and 124243). Additional support for genetic data was provided by the US NIH (U01 DK062370, R01 DK093757, R01 DK072193, and ZIA HG000024). For information about the METSIM study, contact Markku Laakso at markku.laakso@kuh.fi.

**NESCOG (Netherlands Study on Cognition, Environment and Genes)** — This research was part of Science Live, the innovative research program of science center NEMO that enables scientists to carry out real, publishable, peer-reviewed research using NEMO visitors as volunteers.

**NINDS SiGN (The National Institute of Neurological Disorders and Stroke Genetics Network)** — The NINDS International Stroke Genetics Consortium Study dataset was funded by the National Institute of Neurological Disorders and Stroke Cooperative Agreement Award 1U01NS069208. John W. Cole is funded by NIH/NINDS grant number R01 NS114045. The dataset used for the analyses described in this manuscript was obtained from dbGaP at http://www.ncbi.nlm.nih.gov/gap through dbGaP accession number phs000615.

The KORA study, a sub-study within NINDS SiGN, was initiated and financed by the Helmholtz Zentrum München – German Research Center for Environmental Health, which is funded by the German Federal Ministry of Education and Research (BMBF) and by the State of Bavaria. KORA research was supported within the Munich Center of Health Sciences (MC-Health), Ludwig-Maximilians-Universität, as part of LMUinnovativ. Funded by the Bavarian State Ministry of Health and Care through the research project DigiMed Bayern (www.digimed-bayern.de).

**NTR (Netherlands Twin Register) —** NTR would like to gratefully acknowledge the contributions of the participants. This work was supported by the following grants: NIH MH068457-06, 1RC2 MH089951, 1RC2

MH089995, D0042157-01A1, MH081802, DA018673; the Avera Institute for Human Genetics BIOS-consortium NWO-184.021.007, NWO-480-15-001/674; BBMRI–NL: 184.021.007 and 184.033.111, ZonMw 31160008 and NWO 480-15-001/674. D.I. Boomsma acknowledges the Royal Netherlands Academy of Science Professor Award (PAH/6635).

**OMG SCD (Outcome Modifying Genes in Sickle Cell Disease) –** The OMG cohort was funded by NHLBI (R01HL68959, HL79915, HL70769, HL87681). The dataset used for the analyses described in this manuscript was obtained from dbGaP at http://www.ncbi.nlm.nih.gov/gap through dbGaP accession number phs001608.

**OZALC (Australian Twin-Family Studies on Nicotine and Alcohol Genetics)** — OZALC acknowledges the work over many years of staff of the Genetic Epidemiology group at QIMR Berghofer Medical Research Institute (formerly the Queensland Institute of Medical Research) in managing the studies which generated the data used in this analysis. We also acknowledge and appreciate the willingness of study participants to complete multiple, and sometimes lengthy, questionnaires and interviews. Many of the participants were contacted originally through the Australian Twin Registry. Funding for the original studies in which information on alcohol use and smoking status was obtained came from the US National Institutes of Health (AA07535, AA07728, AA11998, AA13320, AA13321, AA14041, AA17688, DA012854 and DA019951); the Australian National Health and Medical Research Council (241944, 339462, 389927, 389875, 389891, 389892, 389938, 442915, 442981, 496739, 552485 and 552498); and the Australian Research Council (A7960034, A79906588, A79801419, DP0770096, DP0212016 and DP0343921). Sarah E. Medland was supported by National Health and Medical Research Council grant APP1172917. Nicholas G. Martin was supported by National Health and Medical Research Council grant APP1172990.

**PAGE (Population Architecture using Genomics and Epidemiology) CARDIA (Coronary Artery Risk Development in Young Adults) –** The Coronary Artery Risk Development in Young Adults Study (CARDIA) is conducted and supported by the National Heart, Lung, and Blood Institute (NHLBI) in collaboration with the University of Alabama at Birmingham (HHSN268201800005I & HHSN268201800007I), Northwestern University (HHSN268201800003I), University of Minnesota (HHSN268201800006I), and Kaiser Foundation Research Institute (HHSN268201800004I). This manuscript has been reviewed by CARDIA for scientific content.

**PAGE (Population Architecture using Genomics and Epidemiology) MEC (Multi-Ethnic Cohort Study) –** The Multi-ethnic Cohort Study was supported by NIH grants U01 CA164973, U01 HG007397, and U01 HG004802. We thank all participants in the Multiethnic Cohort Study.

**SAFS (San Antonio Family Studies) –** Collection of the San Antonio Family Study data was supported in part by National Institutes of Health (NIH) grants R01 HL045522, MH078143, MH078111 and MH083824; and whole genome sequencing of SAFS subjects was supported by U01 DK085524 and R01 HL113323. We are very grateful to the participants of the San Antonio Family Study for their continued involvement in our research programs. The dataset used for the analyses described in this manuscript was obtained from dbGaP at http://www.ncbi.nlm.nih.gov/gap through dbGaP accession number phs001215.

**SardiNIA (SardiNIA project)** — We thank all the volunteers who generously participated in this study and made this research possible. This research was supported by Contracts (HHSN271201600005C, to F.C.) from the Intramural Research, Program of the National Institute on Aging, National Institutes of Health (NIH); (FaReBio2011 'Farmaci e Reti Biotecnologiche di Qualità', to F.C.) from the Italian Ministry of Economy and Finance; a grant (633964, to F.C.) from the Horizon 2020 Research and Innovation Program of the European Union; grants ('Centro per la Ricerca di Nuovi Farmaci per Malattie Rare, Trascurate e della Povertà' and 'Progetto Collezione di Composti Chimici ed Attività di Screening' to F.C.) from Ministero dell'Istruzione, dell'Università e della Ricerca. For information about this study, contact Francesco Cucca (fcucca@irgb.cnr.it).

**SARP (Severe Asthma Research Program) –** The authors thank the SARP participants, investigators, clinical research staff and data coordinating center. SARP was conducted with the support of the National Institutes of Health (NIH), National Heart, Lung, and Blood Institute (NHLBI) grants R01 HL069116, R01 HL069130, R01 HL069149, R01 HL069155, R01 HL069167, R01 HL069170, R01 HL069174, R01 HL069349, U10 HL109086, U10 HL109146, U10 HL109152, U10 HL109164, U10 HL109168, U10 HL109172, U10 HL109250, and U10 HL109257. The dataset used for the analyses described in this manuscript was obtained from dbGaP at http://www.ncbi.nlm.nih.gov/gap through dbGaP accession number phs001446.

**Spit for Science –** Spit for Science has been supported by Virginia Commonwealth University, P20 AA017828, R37AA011408, K02AA018755, P50 AA022537, and K01AA024152 from the National Institute on

Alcohol Abuse and Alcoholism, and UL1RR031990 from the National Center for Research Resources and National Institutes of Health Roadmap for Medical Research. This research was also supported by the National Institute on Drug Abuse of the National Institutes of Health under Award Number U54DA036105 and the Center for Tobacco Products of the U.S. Food and Drug Administration. The content is solely the responsibility of the authors and does not necessarily represent the views of the NIH or the FDA. Data from this study are available to qualified researchers via dbGaP (phs001754.v2.p1). We would like to thank the Spit for Science participants for making this study a success, as well as the many University faculty, students, and staff who contributed to the design and implementation of the project.

The Spit for Science Working Group: Director: Danielle M. Dick, Co-Director: Ananda Amstadter. Registry management: Emily Lilley, Renolda Gelzinis, Anne Morris. Data cleaning and management: Katie Bountress, Amy E. Adkins, Nathaniel Thomas, Zoe Neale, Kimberly Pedersen, Thomas Bannard & Seung B. Cho. Data collection: Amy E. Adkins, Kimberly Pedersen, Peter Barr, Holly Byers, Erin C. Berenz, Erin Caraway, Seung B. Cho, James S. Clifford, Megan Cooke, Elizabeth Do, Alexis C. Edwards, Neeru Goyal, Laura M. Hack, Lisa J. Halberstadt, Sage Hawn, Sally Kuo, Emily Lasko, Jennifer Lend, Mackenzie Lind, Elizabeth Long, Alexandra Martelli, Jacquelyn L. Meyers, Kerry Mitchell, Ashlee Moore, Arden Moscati, Aashir Nasim, Zoe Neale, Jill Opalesky, Cassie Overstreet, A. Christian Pais, Kimberly Pedersen, Tarah Raldiris, Jessica Salvatore, Jeanne Savage, Rebecca Smith, David Sosnowski, Jinni Su, Nathaniel Thomas, Chloe Walker, Marcie Walsh, Teresa Willoughby, Madison Woodroof & Jia Yan. Genotypic data processing and cleaning: Cuie Sun, Brandon Wormley, Brien Riley, Fazil Aliev, Roseann Peterson & Bradley T. Webb.

**THRV (Taiwan Study of Hypertension using Rare Variants) –** The Rare Variants for Hypertension in Taiwan Chinese (THRV) is supported by the National Heart, Lung, and Blood Institute (NHLBI) grants (R01HL111249 and R01HL111249-04S1) in collaboration with Washington University in St. Louis, LA BioMed at Harbor UCLA, University of Texas in Houston, National Health Research Institutes, Taichung Veterans General Hospital, Taipei Veterans General Hospital, Tri-Service General Hospital, National Taiwan University, and Baylor University. THRV is based (substantially) on the parent SAPPHIRe study, along with additional controls. SAPPHIRe was supported by NHLBI grants (U01HL54527, U01HL54498) and Taiwan funds, and the control studies were supported by Taiwan funds. The dataset used for the analyses described in this manuscript was obtained from dbGaP at http://www.ncbi.nlm.nih.gov/gap through dbGaP accession number phs001387.

**UKB (UK Biobank) –** This research has been conducted using the UK Biobank Resource under Application Number 16651. Informed consent was obtained from UK Biobank subjects.

**VTE (Mayo Clinic Venous Thromboembolism Study) –** Individual Propensity to Venous Thrombosis (VTE Candidate Gene study) was funded by NIH (R01HL83141). Risk Factors for Venous Thromboembolism in the Community (Olmsted County VTE study) was funded by NIH (R01HL66216). The EGC gratefully acknowledges the contributions of the participants and of the study staff. The dataset used for the analyses described in this manuscript was obtained from dbGaP at http://www.ncbi.nlm.nih.gov/gap through dbGaP accession number phs001402.

**WGHS (Women's Genome Health Study) –** The Women's Genome Health Study (WGHS) is supported by the National Heart, Lung, and Blood Institute (HL043851, HL080467, and HL099355), the National Cancer Institute (CA047988 and UM1CA182913), with funding for genotyping provided by Amgen. Atrial fibrillation endpoint confirmation was supported by HL-093613 and a grant from the Harris Family Foundation and Watkin's Foundation. The dataset used for the analyses described in this manuscript was obtained from dbGaP at http://www.ncbi.nlm.nih.gov/gap through dbGaP accession number phs001040.

**WHI (Women's Health Initiative)** — The WHI program is funded by the National Heart, Lung, and Blood Institute, National Institutes of Health, U.S. Department of Health and Human Services through contracts 75N92021D00001, 75N92021D00002, 75N92021D00003, 75N92021D00004, 75N92021D00005. The authors thank the WHI investigators and staff for their dedication, and the study participants for making the program possible. A full listing of WHI investigators can be found at: http://www.whi.org/researchers/Documents Write a Paper/WHI Investigator Short List.pdf. For more information about this study, please contact nm9o@nih.gov. The TOPMed dataset used for the analyses described in this manuscript was obtained from dbGaP at http://www.ncbi.nlm.nih.gov/gap through dbGaP accession number phs001237.

**WLS (Wisconsin Longitudinal Study)** — The WLS was funded by the National Institutes for Health and National Institute on Aging (R01 AG041868; R01 AG009775; R01 AG033285; R01 AG060737).

## Individual acknowledgements

Scott Vrieze, Gretchen R.B. Saunders, Seon-Kyeong Jang, Mengzhen Liu, and Jacqueline M. Otto were partially supported by the National Institutes of Health grant numbers R56HG011035, R01DA044283, R01DA042755, and U01DA041120. Dajiang J. Liu, Xingyan Wang, Fang Chen, Chen Wang, Shuang Gao, Bibo Jiang, and Chachrit Khunsriraksakul were partially supported by the National Institutes of Health grant numbers R01GM126479, R56HG011035, R03OD032630, R01HG011035, and R56HG012358. Dajiang J. Liu and Xingyan Wang were in part supported by the Penn State College of Medicine's Biomedical Informatics and Artificial Intelligence Program in the Strategic Plan. Gretchen R.B. Saunders was also funded by National Institutes of Health grant number T32DA050560. Sarah A. Gagliano Taliun was funded by a Junior 1 award from the Fonds de recherche du Québec - Santé (FRQS; https://frq.gouv.qc.ca) and by Operational Funds from the Institut de valorisation des données (IVADO; https://ivado.ca). Eric O. Johnson, Ravi Mathur, and Dana B. Hancock were supported by R01DA042090. Chiara Batini was supported by an internal fellowship at the University of Leicester from the Wellcome Trust Institutional Strategic Support Fund (204801/Z/16/Z) and a UKRI Innovation Fellowship at Health Data Research UK (MR/S003762/1). Andrew W Bergen was partially supported by the National Institute of Health grant number R44AA027675. Computational support was provided by the Minnesota Supercomputing Institute (MSI) at the University of Minnesota.

# List of Supplementary Figures

**Supplementary Figure 1:** Diagram of project workflow.

**Supplementary Figure 2:** Multi-ancestry meta-analysis QQ plots.

**Supplementary Figure 3:** TOPMed reference panel comparisons.

**Supplementary Figure 4:** Replicability Assessment in Trans-Ethnic Studies (RATES) results for 17 independent variants with low posterior probabilities.

**Supplementary Figure 5:** Affinity propagation clustering of correlations between EUR-stratified GWAS meta-analysis results and 1,141 UK Biobank phenotypes.

**Supplementary Figure 1. Diagram of project workflow.** An overview of the primary data and analyses.

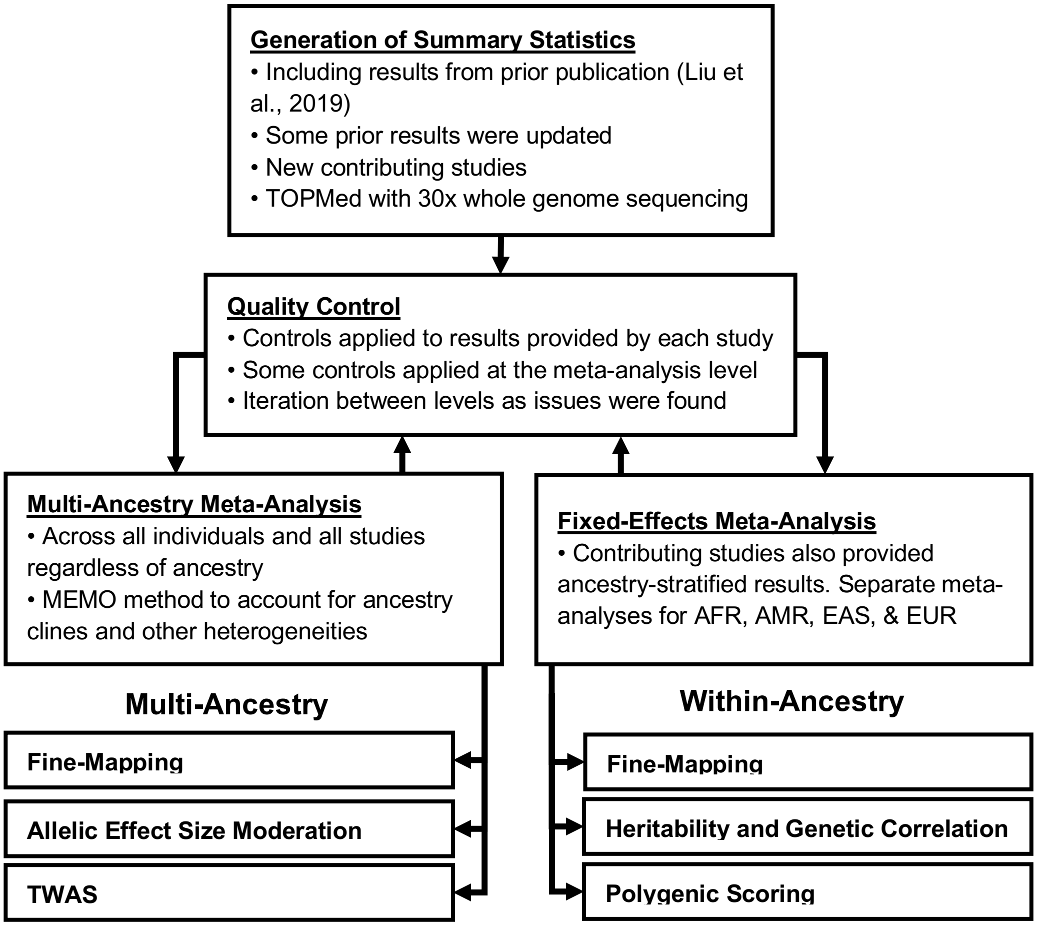

**Supplementary Figure 2. Multi-ancestry meta-analysis QQ plots.** Low frequency variants (.001 < MAF ≤ .01) are shown in red. Common variants (MAF > .01) are shown in black. GC = genomic control. GC correction was applied for low frequency variants only. See Supplementary Note for details on replicability of signals and population stratification.

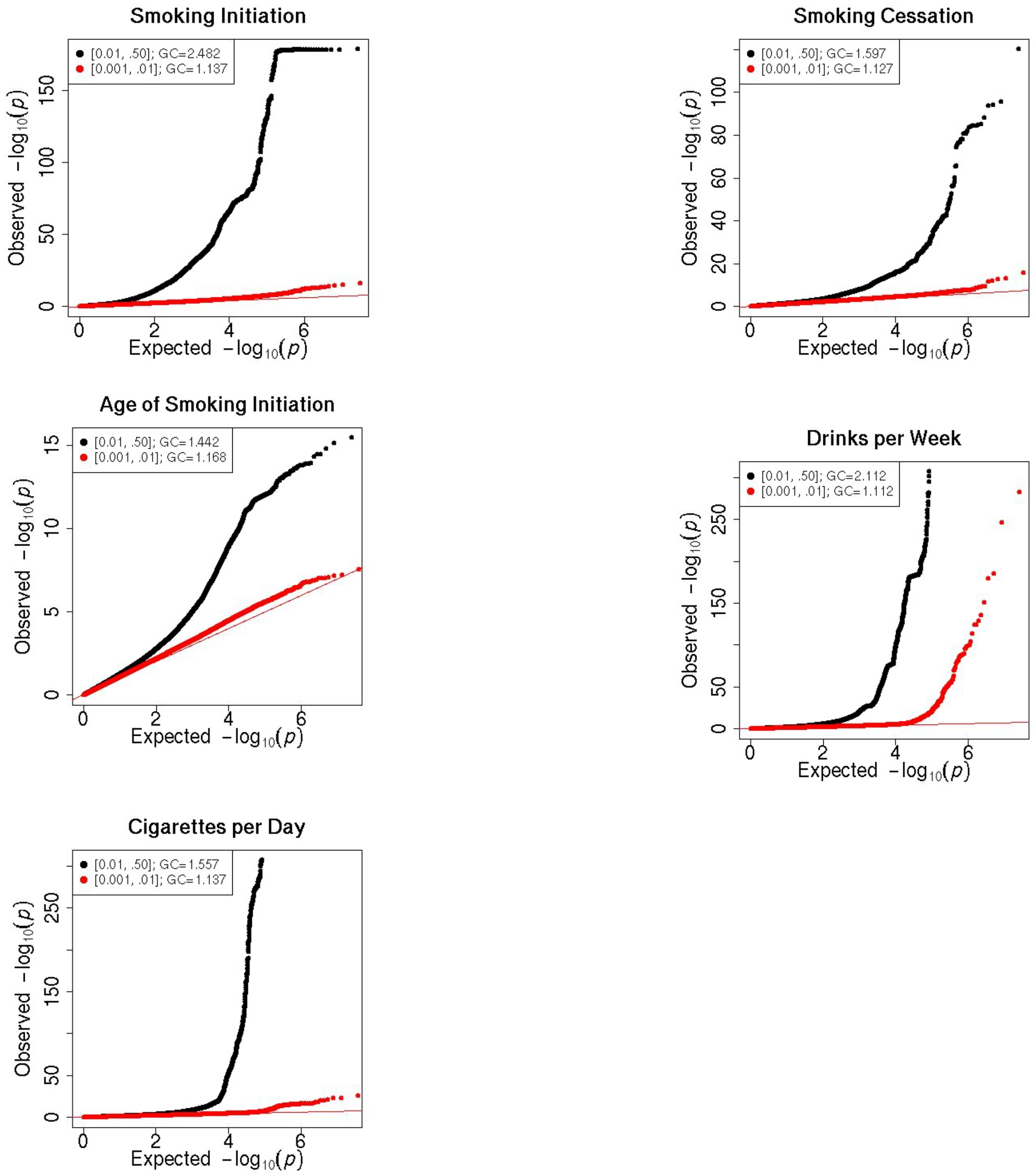

**Supplementary Figure 3. TOPMed reference panel comparisons.** Panel A shows TOPMed principal components (based on individual-level genotypes) projected onto the per-study MDS space (based on allele frequencies from the summary statistics) for components 1-3. TOPMed individuals are shown in color (by assigned ancestry group) with studies (both from TOPMed cohorts [shown as dots] and non-TOPMed cohorts [shown as triangles]) shown in black. The purpose of this is to illustrate the comparability between TOPMed and non-TOPMed ancestry groups. Because of the mapping from the PC to MDS space, the components are not identical to Extended Data Figure 1. The remaining panels show TOPMed reference panel options for an example TOPMed cohort of HCHS_SOL, which is primarily made up of AMR ancestry individuals. Panel B shows where the HCHS_SOL study (black symbol "⊠") exists in the TOPMed PC space for components 1 and 2. All colored points (TOPMed individuals) are identical across panels C-E, with only the black points, representing possible choices for selecting TOPMed individuals to contribute to a reference panel for the HCHS_SOL study, changing. Panel C shows all TOPMed individuals from the HCHS_SOL cohort in black. Panel D shows all TOPMed individuals classified as AMR ancestry in black (this ancestry matched method was used for all TOPMed reference panels). Panel E shows iteratively selected TOPMed reference panel individuals in black. Panel F shows all 1000 Genomes AMR individuals in black.

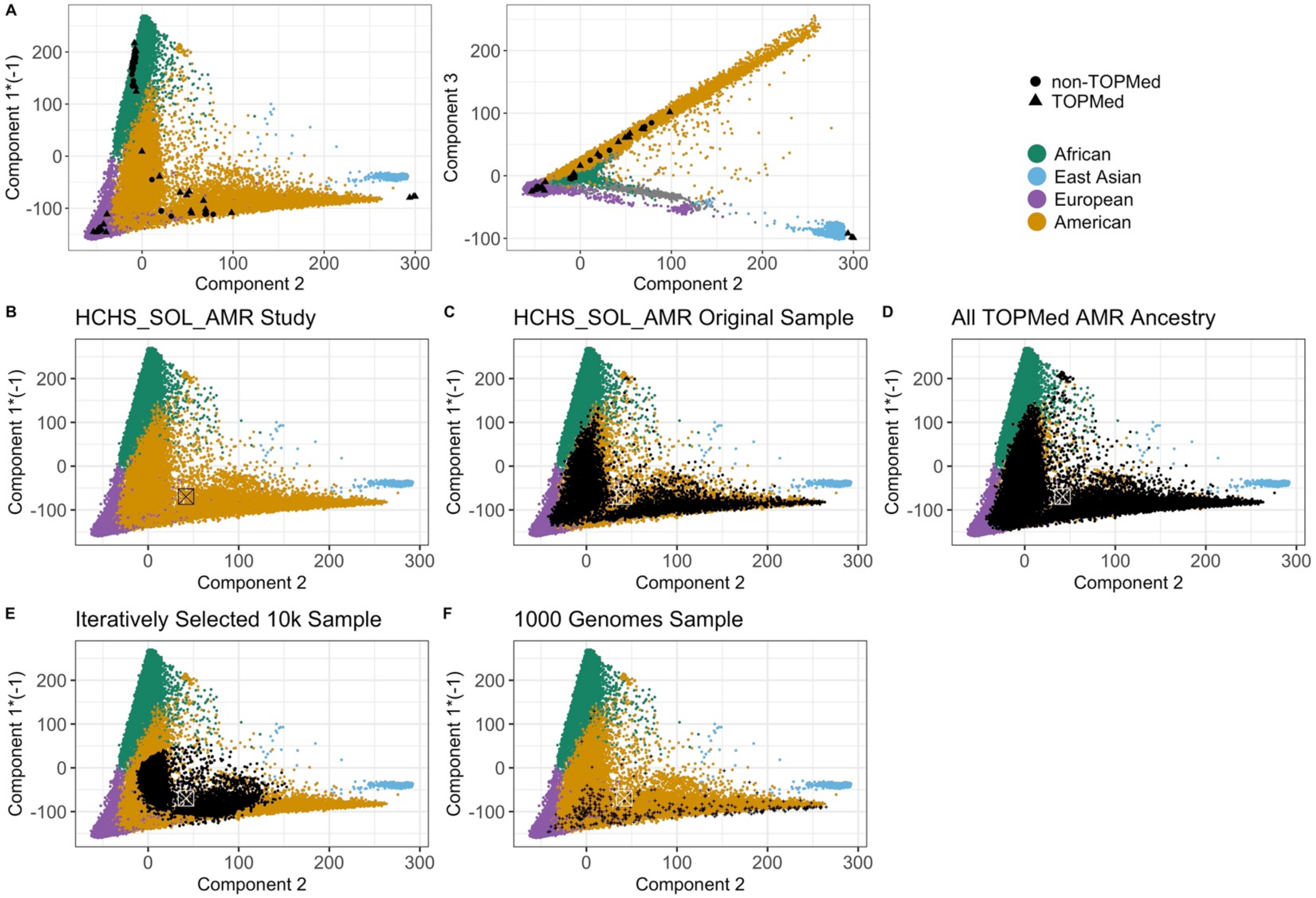

**Supplementary Figure 4. Replicability Assessment in Trans-Ethnic Studies (RATES) results for 17 independent variants with low posterior probabilities.** Each row denotes an independent variant where RATES identified a low posterior probability of a replicable effect in a sufficiently powered study (posterior probability < 0.99). Each variant is listed with its respective posterior probability, two-sided *P*-value from the multi-ancestry meta-analysis, and number of contributing studies. The number of studies in some cases is larger than the total number of cohorts we report due to how summary statistic files were shared (e.g. 23andMe provided summary statistics stratified by sex which will be displayed here as two points/studies but only counted as one cohort) Plots show the variant meta-analytic Z-scores from each contributing study on the x-axis with points jittered on the y-axis for visual clarity. The color denotes the primary ancestry of the cohort; size denotes the cohort sample size. Boxplots are overlayed to highlight outlier cohorts driving the low replicability. Each box denotes the 25th, 50th, and 75th percentiles with whiskers extending to the largest and smallest values with 1.5 times the interquartile range above and below, respectively.

| Phenotype | Variant Info | Z-score Plots |
|---|---|---|
| CigDay | chr2:134785856_A/C<br>Posterior probability = 0.401<br>*P*-value = $1.39\times10^{-9}$<br>Number of studies = 41 | 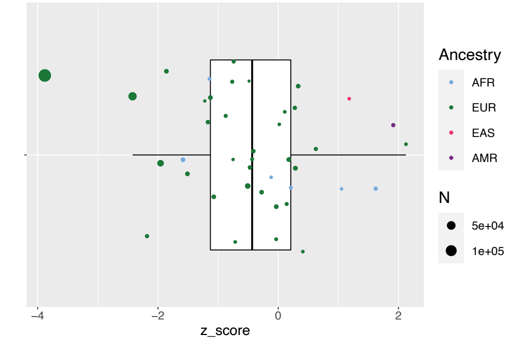 |
| | chr18:45078216_C/T<br>Posterior probability = 0.822<br>*P*-value = $4.93\times10^{-9}$<br>Number of studies = 47 | 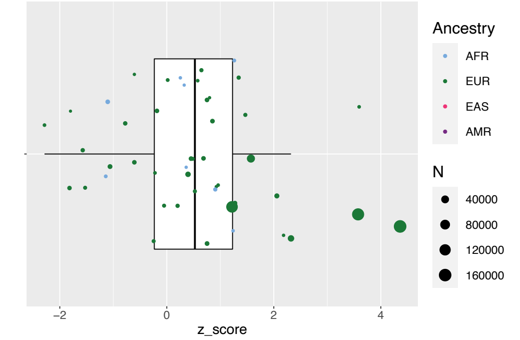 |
| DrnkWk | chr1:102463444_A/G<br>Posterior probability = 0.501<br>*P*-value = $3.27\times10^{-10}$<br>Number of studies = 27 | 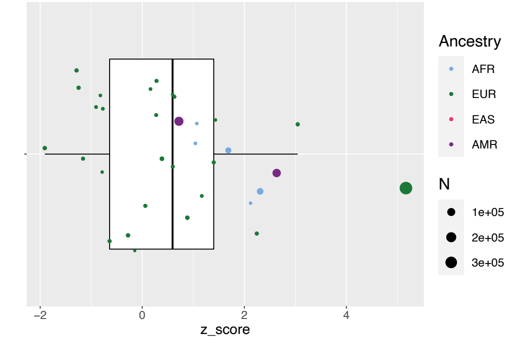 |

<table>
<tr><td rowspan="2"></td><td>chr18:27674354_A/G<br>Posterior probability = 0.973<br>*P*-value = $1.85\times10^{-10}$<br>Number of studies = 60</td><td>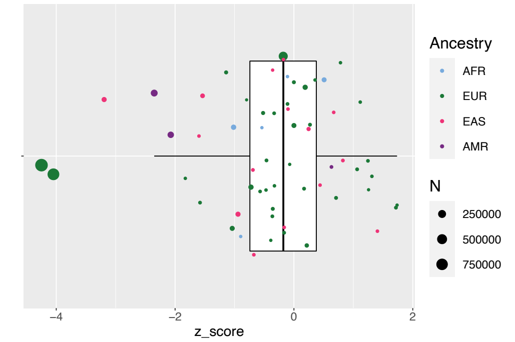
</td></tr>
<tr><td>chr18:43149880_CA/C<br>Posterior probability = 0.887<br>*P*-value = $2.45\times10^{-9}$<br>Number of studies = 7</td><td>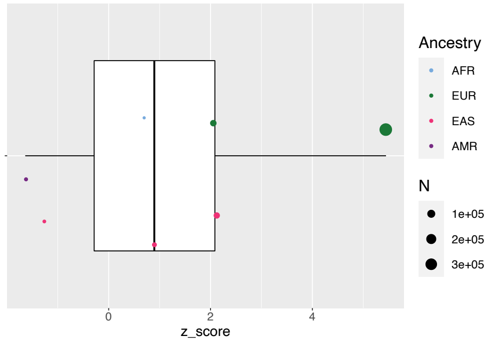
</td></tr>
<tr><td rowspan="3">SmkCes</td><td>chr2:10619084_G/A<br>Posterior probability = 8.02e-260<br>*P*-value = $2.24\times10^{-172}$<br>Number of studies = 38</td><td>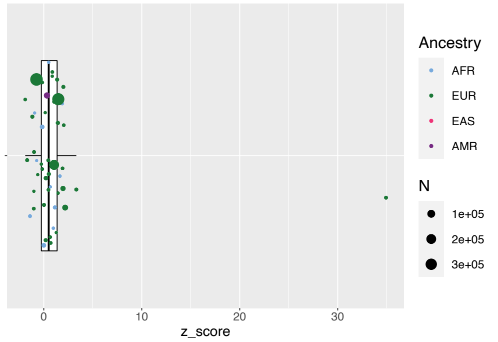
</td></tr>
<tr><td>chr2:160587711_T/A<br>Posterior probability = 4.75e-57<br>*P*-value = $6.49\times10^{-25}$<br>Number of studies = 35</td><td>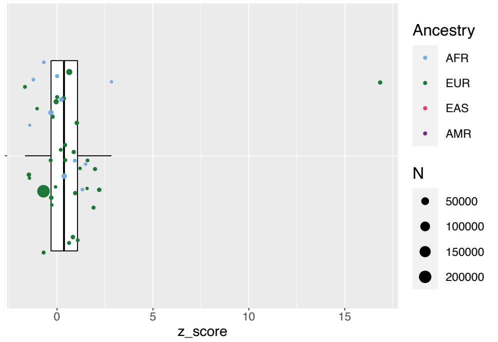
</td></tr>
<tr><td>chr3: 173570664_G/A<br>Posterior probability = 2.39e-92<br>*P*-value = $1\times10^{-51}$<br>Number of studies = 36</td><td>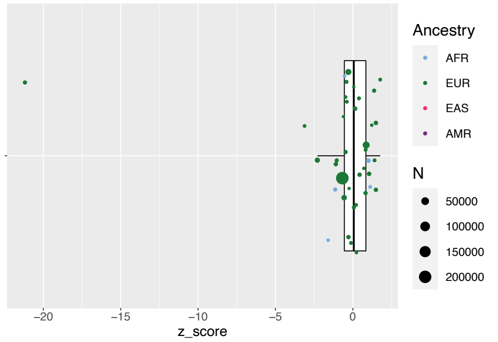
</td></tr>
</table>

| | | |
|---|---|---|
| | chr11: 79227043_T/C<br>Posterior probability = 2.73e-245<br>*P*-value = $1.68 \times 10^{-171}$<br>Number of studies = 38 | 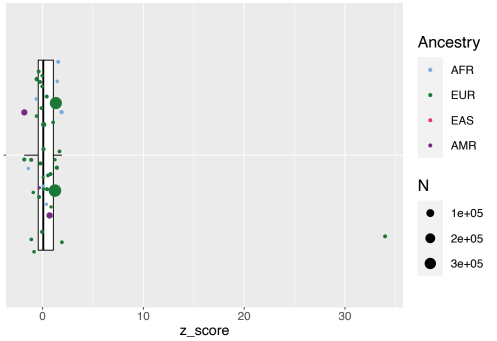 |
| | chr11: 118395808_C/T<br>Posterior probability = 0.989<br>*P*-value = $2.09 \times 10^{-9}$<br>Number of studies = 51 | 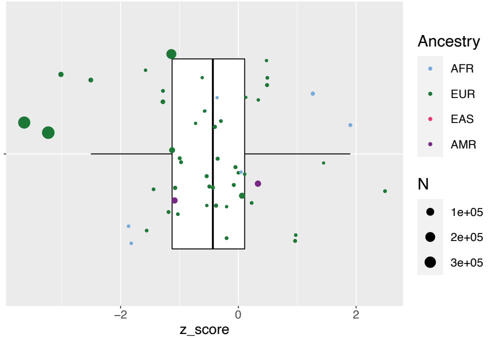 |
| | chr13: 57909623_G/A<br>Posterior probability = 0.985<br>*P*-value = $1.85 \times 10^{-11}$<br>Number of studies = 71 | 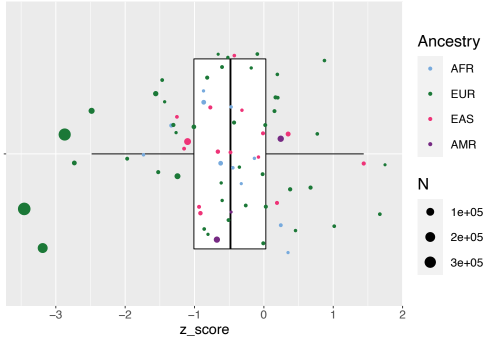 |
| | chr13: 100261629_C/T<br>Posterior probability = 0.0124<br>*P*-value = $2.54 \times 10^{-9}$<br>Number of studies = 73 | 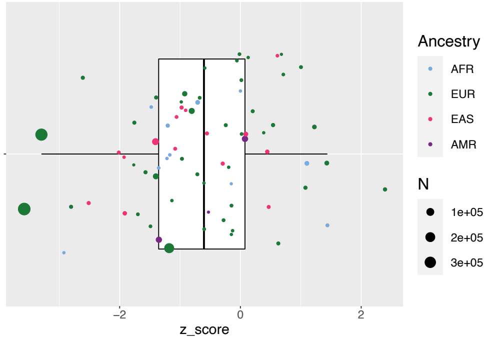 |
| | chr15: 26719238_A/G<br>Posterior probability = 8.02e-85<br>*P*-value = $6.9 \times 10^{-47}$<br>Number of studies = 28 | 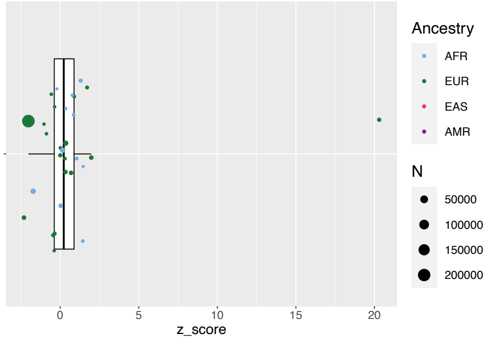 |

| | | |
|---|---|---|
| | chr15: 42576159_T/C<br>Posterior probability = 3.86e-207<br>*P*-value = $7.84\times10^{-142}$<br>Number of studies = 38 | 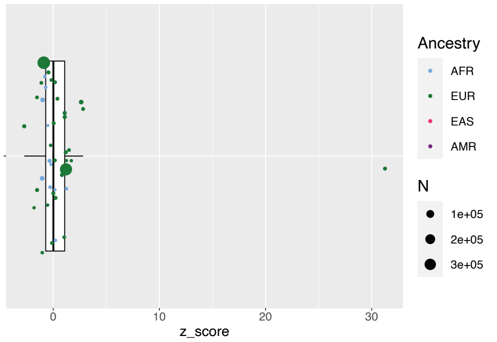 |
| | chr16: 15127835_C/T<br>Posterior probability = 0.0543<br>*P*-value = $1.21\times10^{-9}$<br>Number of studies = 18 | 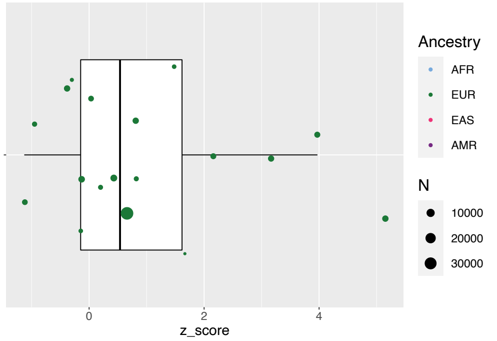 |
| SmkInit | chr3: 38609794_C/T<br>Posterior probability = 0.00545<br>*P*-value = $2.48\times10^{-12}$<br>Number of studies = 5 | 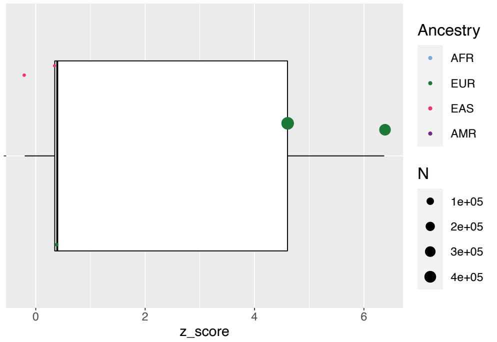 |
| | chr6: 79798179_C/T<br>Posterior probability = 0.0289<br>*P*-value = $3.59\times10^{-13}$<br>Number of studies = 35 | 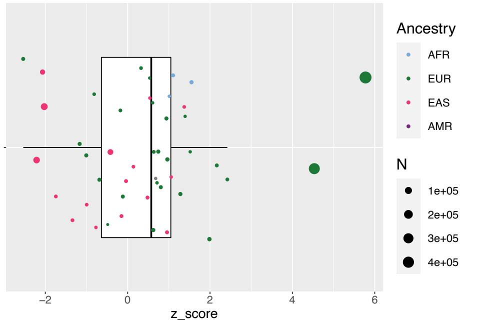 |

**Supplementary Figure 5. Affinity propagation clustering of correlations between EUR-stratified GWAS meta-analysis results and 1,141 UK Biobank phenotypes.** The figure visualizes genetic correlations between UK Biobank phenotypes (rows) and the five smoking/alcohol use phenotypes from the present meta-analyses (columns). Smoking Initiation and Age of Initiation of Smoking show similar patterns of association, as do cigarettes per day and smoking cessation. All the smoking phenotypes show more similar patterns with each other than with drinks per week. The color indicates the absolute magnitude of the correlation.

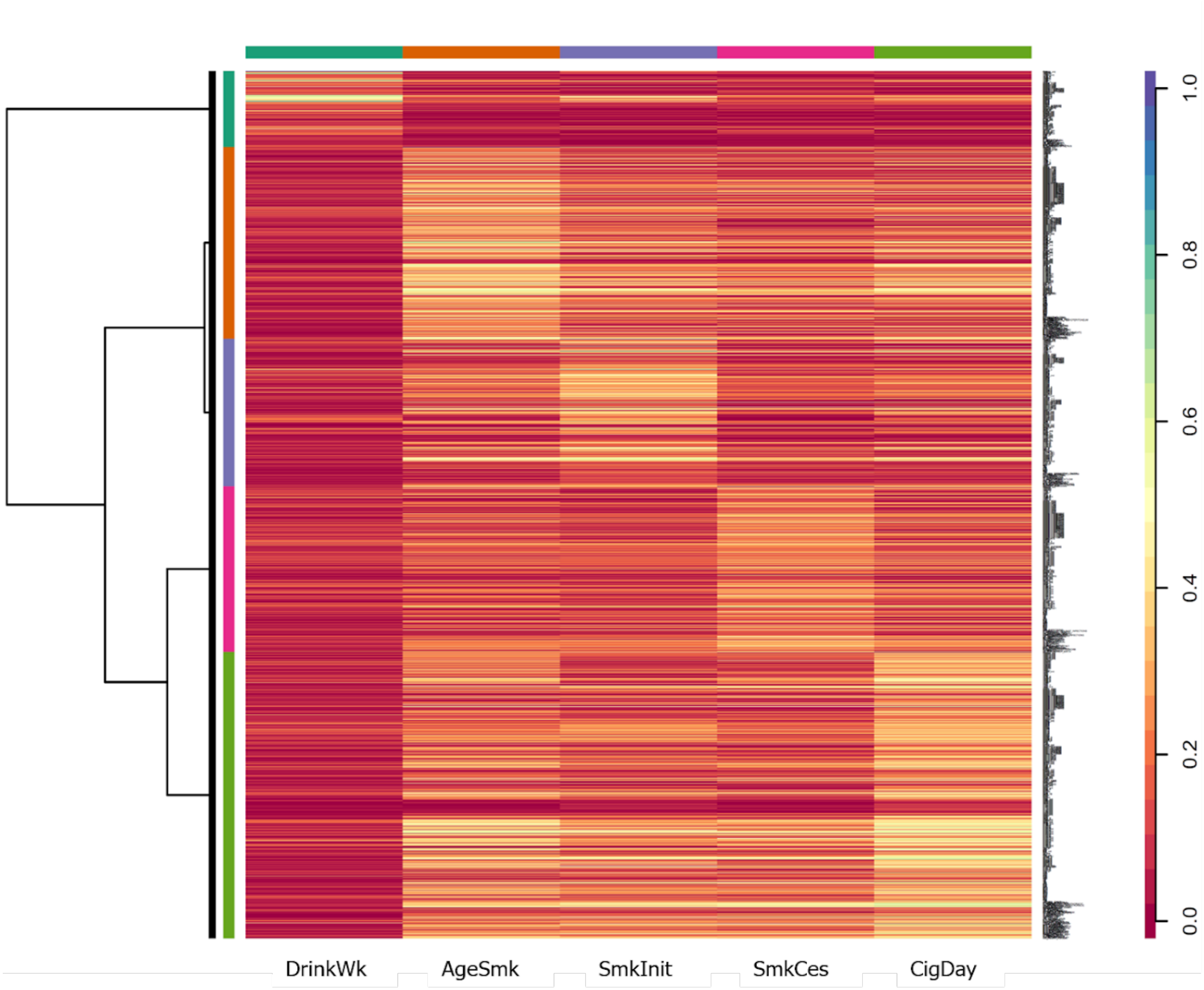

# References


1. Taliun, D. *et al.* Sequencing of 53,831 diverse genomes from the NHLBI TOPMed Program. *Nature* **590**, 290–299 (2021).
2. Liu, M. *et al.* Association studies of up to 1.2 million individuals yield new insights into the genetic etiology of tobacco and alcohol use. *Nature Genetics* **51**, 237–244 (2019).
3. McCarthy, S. *et al.* A reference panel of 64,976 haplotypes for genotype imputation. *Nature Genetics* **48**, 1279–1283 (2016).
4. 1000 Genomes Project Consortium *et al.* A global reference for human genetic variation. *Nature* **526**, 68–74 (2015).
5. Das, S. *et al.* Next-generation genotype imputation service and methods. *Nature Genetics* **48**, 1284–1287 (2016).
6. Bycroft, C. *et al.* The UK Biobank resource with deep phenotyping and genomic data. *Nature* **562**, 203–209 (2018).
7. Huang, J. *et al.* Improved imputation of low-frequency and rare variants using the UK10K haplotype reference panel. *Nature Communications* **6**, 8111 (2015).
8. Howie, B., Fuchsberger, C., Stephens, M., Marchini, J. & Abecasis, G. R. Fast and accurate genotype imputation in genome-wide association studies through pre-phasing. *Nat. Genet.* **44**, 955–959 (2012).
9. Zhan, X., Hu, Y., Li, B., Abecasis, G. R. & Liu, D. J. RVTESTS: an efficient and comprehensive tool for rare variant association analysis using sequence data. *Bioinformatics* **32**, 1423–1426 (2016).
10. Loh, P.-R., Kichaev, G., Gazal, S., Schoech, A. P. & Price, A. L. Mixed-model association for biobank-scale datasets. *Nature Genetics* **50**, 906–908 (2018).
11. Zhou, W. *et al.* Efficiently controlling for case-control imbalance and sample relatedness in large-scale genetic association studies. *Nature Genetics* **50**, 1335–1341 (2018).
12. Cook, J. P., Mahajan, A. & Morris, A. P. Guidance for the utility of linear models in meta-analysis of genetic association studies of binary phenotypes. *Eur J Hum Genet* **25**, 240–245 (2017).
13. Kang, H. M. *et al.* Variance component model to account for sample structure in genome-wide association studies. *Nat Genet* **42**, 348–354 (2010).
14. Xue, A. *et al.* Genome-wide association analyses identify 143 risk variants and putative regulatory mechanisms for type 2 diabetes. *Nat Commun* **9**, 2941 (2018).
15. Fuchsberger, C. *et al.* The genetic architecture of type 2 diabetes. *Nature* **536**, 41–47 (2016).
16. Chang, C. C. *et al.* Second-generation PLINK: rising to the challenge of larger and richer datasets. *Gigascience* **4**, 7 (2015).
17. Abdellaoui, A. *et al.* Population structure, migration, and diversifying selection in the Netherlands. *European Journal of Human Genetics* **21**, 1277–1285 (2013).
18. Yang, J. *et al.* Genomic inflation factors under polygenic inheritance. *European Journal of Human Genetics* **19**, 807–812 (2011).
19. Gibbs, R. A. *et al.* The International HapMap Project. *Nature* **426**, 789–796 (2003).
20. Zhang, D., Dey, R. & Lee, S. Fast and robust ancestry prediction using principal component analysis. *Bioinformatics* **36**, 3439–3446 (2020).
21. Jiang, Y. *et al.* Proper conditional analysis in the presence of missing data: Application to large scale meta-analysis of tobacco use phenotypes. *PLOS Genetics* **14**, e1007452 (2018).
22. Yang, J. *et al.* Conditional and joint multiple-SNP analysis of GWAS summary statistics identifies additional variants influencing complex traits. *Nat Genet* **44**, 369-S3 (2012).
23. Atkinson, E. G. *et al.* Tractor uses local ancestry to enable the inclusion of admixed individuals in GWAS and to boost power. *Nat Genet* **53**, 195–204 (2021).
24. Atkinson, E. G. *et al.* Reply to: On powerful GWAS in admixed populations. *Nat Genet* **53**, 1634–1635 (2021).
25. Hou, K., Bhattacharya, A., Mester, R., Burch, K. S. & Pasaniuc, B. On powerful GWAS in admixed populations. *Nat Genet* **53**, 1631–1633 (2021).
26. McGuire, D. *et al.* Model-based assessment of replicability for genome-wide association meta-analysis. *Nat Commun* **12**, 1964 (2021).

27. Bulik-Sullivan, B. K. *et al.* LD Score regression distinguishes confounding from polygenicity in genome-wide association studies. *Nature Genetics* **47**, 291–295 (2015).
28. Luo, Y. *et al.* Estimating heritability and its enrichment in tissue-specific gene sets in admixed populations. *Human Molecular Genetics* **30**, 1521–1534 (2021).
29. Luo, Y. *et al.* Estimating heritability and its enrichment in tissue-specific gene sets in admixed populations. *bioRxiv* 503144 (2020) doi:10.1101/503144.
30. Lee, J. J., McGue, M., Iacono, W. G. & Chow, C. C. The accuracy of LD Score regression as an estimator of confounding and genetic correlations in genome-wide association studies. *Genet Epidemiol* **42**, 783–795 (2018).
31. Okbay, A. *et al.* Genetic variants associated with subjective well-being, depressive symptoms, and neuroticism identified through genome-wide analyses. *Nature Genetics* **48**, 624–633 (2016).
32. Lee, J. J. *et al.* Gene discovery and polygenic prediction from a genome-wide association study of educational attainment in 1.1 million individuals. *Nature Genetics* **50**, 1112–1121 (2018).
33. Zaitlen, N., Paşaniuc, B., Gur, T., Ziv, E. & Halperin, E. Leveraging Genetic Variability across Populations for the Identification of Causal Variants. *The American Journal of Human Genetics* **86**, 23–33 (2010).
34. Chen, J. *et al.* The trans-ancestral genomic architecture of glycemic traits. *Nat Genet* **53**, 840–860 (2021).
35. Bryois, J. *et al.* Genetic Identification of Cell Types Underlying Brain Complex Traits Yields Insights Into the Etiology of Parkinson's Disease. *Nat Genet* **52**, 482–493 (2020).
36. Lonsdale, J. *et al.* The Genotype-Tissue Expression (GTEx) project. *Nat Genet* **45**, 580–585 (2013).
37. Gamazon, E. R. *et al.* A gene-based association method for mapping traits using reference transcriptome data. *Nat Genet* **47**, 1091–1098 (2015).
38. Liu, Y. & Xie, J. Cauchy combination test: a powerful test with analytic p-value calculation under arbitrary dependency structures. *J Am Stat Assoc* **115**, 393–402 (2020).
39. Horn, J. L. A rationale and test for the number of factors in factor analysis. *Psychometrika* **30**, 179–185 (1965).
40. Leeuw, C. A. de, Mooij, J. M., Heskes, T. & Posthuma, D. MAGMA: Generalized Gene-Set Analysis of GWAS Data. *PLOS Computational Biology* **11**, e1004219 (2015).
41. Gene Ontology Consortium. The Gene Ontology resource: enriching a GOld mine. *Nucleic Acids Res* **49**, D325–D334 (2021).
42. Frey, B. J. & Dueck, D. Clustering by Passing Messages Between Data Points. *Science* **315**, 972–976 (2007).
43. Hernán, M. A., Hernández-Díaz, S. & Robins, J. M. A structural approach to selection bias. *Epidemiology* **15**, 615–625 (2004).
44. Gollust, S. E. *et al.* Consumer Perspectives on Access to Direct-to-Consumer Genetic Testing: Role of Demographic Factors and the Testing Experience. *The Milbank Quarterly* **95**, 291–318 (2017).
45. Roberts, J. S. *et al.* Direct-to-Consumer Genetic Testing: User Motivations, Decision Making, and Perceived Utility of Results. *PHG* **20**, 36–45 (2017).
46. Pirastu, N. *et al.* Genetic analyses identify widespread sex-differential participation bias. *Nat Genet* **53**, 663–671 (2021).
47. Harris, K. M. *et al.* Cohort Profile: The National Longitudinal Study of Adolescent to Adult Health (Add Health). *Int J Epidemiol* **48**, 1415–1415k (2019).
48. Vilhjálmsson, B. J. *et al.* Modeling Linkage Disequilibrium Increases Accuracy of Polygenic Risk Scores. *The American Journal of Human Genetics* **97**, 576–592 (2015).
49. Lee, S. H., Goddard, M. E., Wray, N. R. & Visscher, P. M. A better coefficient of determination for genetic profile analysis. *Genet Epidemiol* **36**, 214–224 (2012).
50. Matoba, N. *et al.* GWAS of 165,084 Japanese individuals identified nine loci associated with dietary habits. *Nat Hum Behav* **4**, 308–316 (2020).
