# Supplementary material for: Genetic diversity fuels gene discovery for tobacco and alcohol use
Source: Nature. 2022 Dec 7;612(7941):720–4. doi: 10.1038/s41586-022-05477-4 (PMC9771818; doi:10.1038/s41586-022-05477-4)
Supplement: Supplementary file 2 — Reporting Summary [file 41586_2022_5477_MOESM2_ESM.pdf]

nature portfolio

Corresponding author(s): Dajiang Liu
Scott Vrieze

Last updated by author(s): Oct 14, 2022

Reporting Summary

Nature Portfolio wishes to improve the reproducibility of the work that we publish. This form provides structure for consistency and transparency in reporting. For further information on Nature Portfolio policies, see our Editorial Policies and the Editorial Policy Checklist.

Statistics

For all statistical analyses, confirm that the following items are present in the figure legend, table legend, main text, or Methods section.

n/a	Confirmed	
☐	☒	The exact sample size (n) for each experimental group/condition, given as a discrete number and unit of measurement
☐	☒	A statement on whether measurements were taken from distinct samples or whether the same sample was measured repeatedly
☐	☒	The statistical test(s) used AND whether they are one- or two-sided<br>*Only common tests should be described solely by name; describe more complex techniques in the Methods section.*
☐	☒	A description of all covariates tested
☐	☒	A description of any assumptions or corrections, such as tests of normality and adjustment for multiple comparisons
☐	☒	A full description of the statistical parameters including central tendency (e.g. means) or other basic estimates (e.g. regression coefficient) AND variation (e.g. standard deviation) or associated estimates of uncertainty (e.g. confidence intervals)
☐	☒	For null hypothesis testing, the test statistic (e.g. F, t, r) with confidence intervals, effect sizes, degrees of freedom and P value noted<br>*Give P values as exact values whenever suitable.*
☐	☒	For Bayesian analysis, information on the choice of priors and Markov chain Monte Carlo settings
☒	☐	For hierarchical and complex designs, identification of the appropriate level for tests and full reporting of outcomes
☐	☒	Estimates of effect sizes (e.g. Cohen's d, Pearson's r), indicating how they were calculated

Our web collection on statistics for biologists contains articles on many of the points above.

Software and code

Policy information about availability of computer code

Data collection	No software was used.
Data analysis	All studies used either ShapeIt2 or EAGLEv2.4 to phase genotypes and used either Minimac3 or IMPUTE2 for imputation. Summary statistics were generated using RVTESTS release v1.9.7 or v1.9.9, BOLT-LMM v2.3.2, or SAIGE v0.35.8.1. Standard quality control used PLINK v1.9 or v2.0, BCFtools v1.9, and VCFtools v0.1.16. Meta-analyses and conditional analyses were performed using rareGWAMA v0.7 in R v3.6.0 and GCTA v1.93.0 and v1.94.0. Transcriptome-wide association analysis was performed using TESLA (implemented in rareGWAMA). Replicability of associated loci was performed using RATES v1.0.0. Standard and covariate-adjusted LD Score Regression was used to measure heritability, test for population stratification, and estimate genetic correlations (LDSC v1.0.1 and cov-LDSC v1.0.0). LDpred v1.0.8 was used to construct the polygenic scores.

For manuscripts utilizing custom algorithms or software that are central to the research but not yet described in published literature, software must be made available to editors and reviewers. We strongly encourage code deposition in a community repository (e.g. GitHub). See the Nature Portfolio guidelines for submitting code & software for further information.

Data

Policy information about availability of data

All manuscripts must include a data availability statement. This statement should provide the following information, where applicable:

- Accession codes, unique identifiers, or web links for publicly available datasets
- A description of any restrictions on data availability
- For clinical datasets or third party data, please ensure that the statement adheres to our policy

GWAS summary statistics can be downloaded online (https://doi.org/10.13020/przg-dp88) with more information available here: https://genome.psych.umn.edu/

index.php/GSCAN. We provide association results for variants that passed quality-control filters in the multi-ancestry and ancestry-stratified results for each of the five substance use phenotypes, excluding data provided by 23andMe. Ancestry-stratified polygenic score weights based on ancestry-stratified summary statistics are also provided. 23andMe results are available directly from the company.

Field-specific reporting

Please select the one below that is the best fit for your research. If you are not sure, read the appropriate sections before making your selection.

☒ Life sciences ☐ Behavioural & social sciences ☐ Ecological, evolutionary & environmental sciences

For a reference copy of the document with all sections, see nature.com/documents/nr-reporting-summary-flat.pdf

Life sciences study design

All studies must disclose on these points even when the disclosure is negative.

Sample size	No sample size calculation was necessary as we increased our sample size to the extent possible. We contacted as many studies (with our phenotypes of interest) as possible and applied for relevant studies available in public repositories. Our meta-analysis includes the largest sample size of similar phenotypes to date and therefore, our results are sufficiently powered.
Data exclusions	We excluded results from smaller studies when those results behaved unusually (e.g., inflated or deflated genomic controls), and there was no alternative explanation (e.g., inflation was due to polygenic signal). We applied filters to the genomic data post meta-analysis (minor allele frequency > .1% for European-stratified results or > 1% for all others, effective sample size of at least 1% per phenotype and at least 3 studies must be included for each variant) in order to only report variants on which we had robust results. Finally, we removed 17 loci in which the lead SNPs posterior probability of replicability fell below a threshold of .99, although these are reported in the supplementary materials.
Replication	In order to maximize power to detect the variants, we did not separate our sample into a separate discovery and replication set. We used a trans-ancestry extension of the Meta-Analysis Model-based Assessment for replicability (MAMBA) to assess the posterior probability of replicability of associations without an independent replication sample. References are available in the manuscript.
Randomization	N/A. No randomization was employed as the current study was observational and used all available participants.
Blinding	N/A. Blinding was not applicable to the current study as we did not employ any intervention.

Reporting for specific materials, systems and methods

We require information from authors about some types of materials, experimental systems and methods used in many studies. Here, indicate whether each material, system or method listed is relevant to your study. If you are not sure if a list item applies to your research, read the appropriate section before selecting a response.

Materials & experimental systems

n/a	Involved in the study	
☒	☐	Antibodies
☒	☐	Eukaryotic cell lines
☒	☐	Palaeontology and archaeology
☒	☐	Animals and other organisms
☐	☒	Human research participants
☒	☐	Clinical data
☒	☐	Dual use research of concern

Methods

n/a	Involved in the study	
☒	☐	ChIP-seq
☒	☐	Flow cytometry
☒	☐	MRI-based neuroimaging

Human research participants

Policy information about studies involving human research participants

Population characteristics	All participants were adults. We included all available individuals of all genders and sexes from European, African, American, or East Asian ancestry populations.
Recruitment	We did not do any recruitment. Analysis was of existing de-identified data.
Ethics oversight	University of Minnesota IRB

Note that full information on the approval of the study protocol must also be provided in the manuscript.
